# An Anti-BCMA Affibody Affinity Protein for Therapeutic and Diagnostic Use in Multiple Myeloma

**DOI:** 10.3390/ijms26115186

**Published:** 2025-05-28

**Authors:** Kim Anh Giang, Johan Nilvebrant, Hao Liu, Harpa Káradóttir, Yumei Diao, Stefan Svensson Gelius, Per-Åke Nygren

**Affiliations:** 1 Department of Protein Science, KTH-Royal Institute of Technology, SE-114 28 Stockholm, Sweden; kagiang@kth.se (K.A.G.); johanni@kth.se (J.N.); lhhot150@gmail.com (H.L.); 2 Oncopeptides AB, SE-171 48 Solna, Sweden; harpak91@gmail.com (H.K.); yumei.diao@oncopeptides.com (Y.D.); stefan.svensson.gelius@oncopeptides.com (S.S.G.)

**Keywords:** affibody, BCMA, multiple myeloma

## Abstract

B Cell Maturation Antigen (BCMA) has gained considerable attention as a target in directed therapies for multiple myeloma (MM) treatment, via immunoglobulin-based bispecific T cell engagers or CAR T cell strategies. We describe the development of alternative, non-immunoglobulin BCMA-recognising affinity proteins, based on the small (58 aa) three-helix bundle affibody scaffold. A first selection campaign using a naïve affibody phage library resulted in the isolation of several BCMA-binding clones with different kinetic profiles. One clone showing the slowest dissociation kinetics was chosen as the template for the construction of two second-generation libraries. Characterization of output clones from selections using these libraries led to the identification of clone 1-E6, which demonstrated low nM affinity to BCMA and high thermal stability. Biosensor experiments showed that 1-E6 interfered with the binding of BCMA to both its natural ligand APRIL and to the clinically evaluated anti-BCMA monoclonal antibody belantamab, suggesting overlapping epitopes. A fluorescently labelled head-to-tail homodimer construct of 1-E6 showed specific binding to the BCMA^+^ MM.1s cell line in both flow cytometry and fluorescence microscopy. Taken together, the results suggest that the small anti-BCMA affibody 1-E6 could be an interesting alternative to antibody-based affinity units in the development of BCMA-targeted therapies and diagnostics.

## 1. Introduction

Multiple myeloma (MM) is a cancer of plasma cells in the bone marrow (BM), which can manifest as bone pain, anaemia, BM failure, renal impairment, and a higher incidence of infections [1]. The long-term remission and survival rates in MM patients have improved during the past two decades due to advances in treatment development, largely credited to the clinical approvals of targeted immunotherapies. These include monoclonal antibodies (mAbs), bispecific antibodies (bsAbs) redirecting T cells, and CAR T cell therapy. The affinity proteins used as the targeting moieties in these immunotherapies are typically based on antibodies or antibody fragments (mainly scFvs) [1,2,3]. Additional antibody-based immunotherapies for MM treatment are currently in clinical trials with promising results [4,5,6,7]. However, MM is still considered an incurable disease, and further development of novel immunotherapies involving tumour target-recognition units is warranted [4].

For such development, alternative classes of non-immunoglobulin affinity proteins may be considered. These proteins differ from antibody-based affinity proteins in e.g., their size, structural organisation, and engineering possibilities, which could be advantageous when used as target recognition units in biologicals [8,9]. Affibodies are a class of non-immunoglobulin affinity proteins with a small size (6.5–7 kDa) and a minimalist architecture consisting of three alpha helices [10,11]. Using combinatorial protein engineering principles, in which typically 13–15 surface-located positions are simultaneously randomised to create libraries for in vitro selection using e.g., phage or cell display, high-affinity affibody affinity proteins binding to diverse targets can be isolated [11,12]. Examples of clinically relevant targets addressed by affibodies include HER2, EGFR, PD-L1 and IL-17A, with demonstrated utility in in vivo tumour imaging, fluorescence-guided surgery, patient stratification in immuno-oncology, and autoimmune disease prevention, respectively [13,14,15,16].

B Cell Maturation Antigen (BCMA) is a target of considerable interest in MM [17]. Overexpression of BCMA on malignant plasma cells is associated with the proliferation and survival of these cells, via activation by its natural ligands: A Proliferation Inducing Ligand (APRIL) and B cell Activating Factor (BAFF), of which APRIL is the preferred ligand [18,19,20]. Several clinically approved immunotherapies are directed to BCMA, including the CAR T cell therapies idecabtagene vicleucel (Abecma) and ciltacabtagene autoleucel (Carvykti), and the BCMA x CD3 bsAbs teclistamab (Tecvayli) and elranatamab (Elrexfio) [21].

In the present study, we describe the development of affibody affinity proteins targeting BCMA for potential application as a tumour-recognition unit in immunotherapeutic agents and as a diagnostic tool for patient stratification based on BCMA expression. We describe the use of phage display for the isolation of BCMA-targeting affibodies from naïve and second-generation libraries. The main candidate clone denoted 1-E6 was characterised in detail using binding kinetics analyses, circular dichroism spectroscopy and competitive binding measurements with the BCMA binding proteins APRIL and the clinically evaluated anti-BCMA mAb belantamab. Binding to BCMA-expressing cells was investigated using flow cytometry and fluorescence microscopy. Taken together, the results suggest that the developed anti-BCMA affibody 1-E6 has the potential to be used as a BCMA-targeting unit in reagents aimed for use in both therapeutic and diagnostic applications in MM.

## 2. Results

### 2.1. Initial Identification and Characterisation of First-Generation BCMA-Binding Affibodies

**Figure 1:**
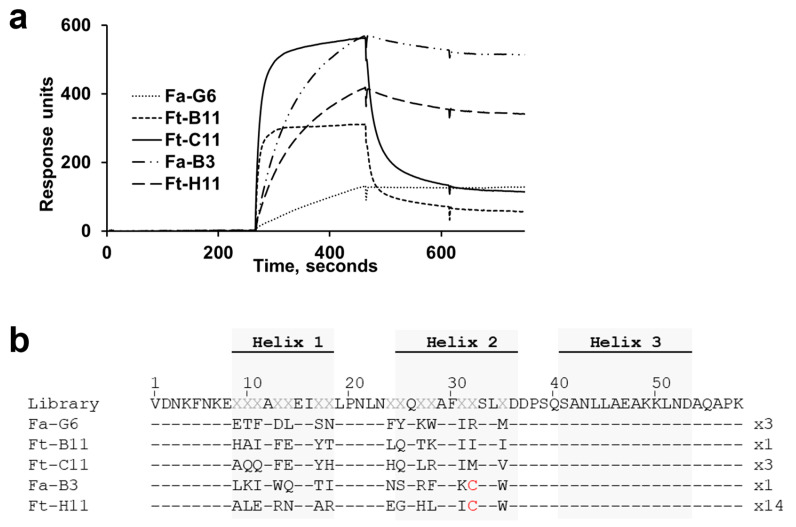
**First-generation anti-B Cell Maturation Antigen (BCMA) affibodies.** (**a**) Overlay of single 200 nM concentration surface plasmon resonance (SPR) sensorgrams for five recombinantly expressed BCMA-targeting affibody candidates (His_6_-affibody-ABD format), binding to immobilised human BCMA-rFc. (**b**) Amino acid sequence alignment of the five clones, showing the amino acid occupancies in the variable positions, compared to the library gene (variable positions denoted X in the library gene sequence). Note the cysteines (red) in position 32 in clones Fa-B3 and Ft-H11. The numbers above the alignment indicate the amino acid residue numbering of the affibody 58-amino-acid scaffold sequence. The number to the right of each sequence indicates its frequency of appearance during the sequencing of 54 ELISA-positive candidates.

To obtain affibody binders to human BCMA, a large (3 × 10^10^) phage display affibody library [22] was used for selections against biotinylated recombinant human BCMA extracellular domain (ECD) (residues 5–50 of human BCMA, containing the ECD), fused to the Fc portion of rabbit IgG (BCMA-rFc). Four cycles of selection were performed in three parallel tracks. These successive selection cycles and parallel tracks varied in target antigen concentration (50–150 nM), selection incubation time, total washing time, and in elution strategy (low pH or trypsin cleavage). After the fourth selection cycle, 96 output clones from each track were evaluated for binding to human BCMA-rFc by monoclonal phage ELISA (Figure S1) followed by DNA sequencing. Twenty-five unique and ELISA-positive clones were subcloned for soluble expression in *Escherichia coli* with an N-terminal hexahistidine (His_6_) tag and a C-terminal 5 kDa ABD (albumin binding domain) fusion partner. IMAC-purified His_6_-affibody-ABD fusion proteins were then analysed using surface plasmon resonance (SPR) at a single 200 nM concentration for binding to BCMA-rFc. An overlay of the resulting SPR sensorgrams for the five variants, denoted Fa-G6, Ft-B11, Ft-C11, Fa-B3 and Ft-H11, which showed either the highest binding responses or slowest off-rates, is shown in Figure 1a. Variants Ft-C11 and Ft-B11 both demonstrated fast binding kinetics (both association and dissociation), whereas variants Fa-G6, Fa-B3 and Ft-H11 showed much slower dissociation kinetics, indicating the formation of highly stable complexes with BCMA.

Interestingly, the sequences of variants Fa-B3 and Ft-H11 both contained a cysteine in the randomised position 32 (Figure 1b), implying that the slow dissociation observed could be due to avidity effects from homodimer formation via disulfide (S-S) bridges. As the library gene design did not include cysteines, the appearance of cysteine residues could possibly be due to library gene synthesis or PCR amplification errors — or random mutations occurring during the selection process. To further investigate the significance of these cysteine residues, cysteine-to-serine mutation variants (C32S) of Fa-B3 and Ft-H11 were produced. SDS-PAGE analysis under non-reducing and reducing conditions confirmed the presence of S-S-bridged homodimers in the cysteine-containing clones, while the C32S mutants demonstrated presence of only monomers (Figure S2a). Analysis by SPR showed that binding to BCMA was lost upon mutating the cysteine residue to a serine in both Fa-B3 and Ft-H11(Figure S2b). The cysteine-containing clones Fa-B3 and Ft-H11 were therefore excluded from further evaluation in this study (see 3. Discussion).

### 2.2. Circular Dichroism (CD) Spectroscopy Studies

In order to assess the secondary structure content and thermal stability of the affibodies using CD spectroscopy, the three candidate binders Fa-G6, Ft-B11, Ft-C11 were subcloned and purified as N-terminally His_6_-tagged constructs, without their previously included ABD fusion partner. Analysis of the ellipticity at wavelengths ranging 195–260 nm showed an expected high alpha-helical secondary structure content for all three affibodies, and all variants showed ability to fully or almost fully refold after thermal denaturation (Figure S3a). The melting temperatures (Tm) were measured by recording the ellipticity at 221 nm, while heating the affibodies from 20 °C to 90 °C (5 °C/min). The resulting thermal denaturation profiles corresponded to Tm values of approximately 49 °C for Ft-B11 and 52 °C for Fa-G6. The irregular melting curve profile for Ft-C11 suggested biphasic melting behaviour, with one phase between 20–50 °C and a second phase between 50–90 °C, thus yielding the Tm values 36 °C and 64 °C for these phases, respectively (Figure S3b,c). Based on these stability analyses and the previously observed slow off-rate kinetics, the Fa-G6 variant appeared as the most promising variant to bring forward.

### 2.3. Alanine Scanning

**Figure 2:**
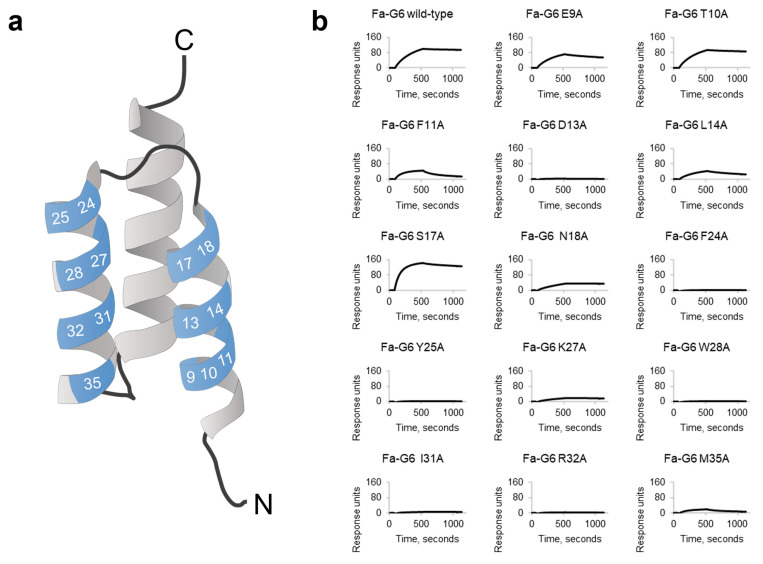
**Alanine scanning of the candidate BCMA-binding clone Fa-G6.** (**a**) The amino acid residues in the 14 variable positions (blue) located on the first two helices of the Fa-G6 clone were individually substituted to alanine. The numbers correspond to the amino acid residue numbering of the affibody 58-amino-acid scaffold sequence, from the N-terminus (N) to the C-terminus (C). The image is based on 5U4Y.pdb. (**b**) Single concentration (200 nM) SPR sensorgrams of the 14 alanine variants binding to immobilised human BCMA-rFc, compared to the Fa-G6 wild-type clone.

To possibly improve the binding affinity and stability further, we proceeded with the construction of a second-generation library based on the Fa-G6 variant. To guide the design of this library, Fa-G6 was subjected to an alanine scanning experiment in which the residue in each variable position was mutated to alanine (Figure 2a), to investigate their importance for BCMA binding and protein stability. To this end, 14 alanine variants of Fa-G6 as well as the Fa-G6 wild-type clone were produced in *E. coli* and screened for binding to BCMA-rFc using SPR. The resulting sensorgrams (Figure 2b) showed that alanine substitutions in position 13 in the first helix and in all variable positions located in the second helix (positions 24, 25, 27, 28, 31, 32 and 35) completely or almost completely removed the binding to BCMA. The BCMA binding was also affected by some alanine substitutions in helix 1 (positions 11, 14 and 18), but to a lesser extent. Interestingly, substitution to alanine in position 17 seemed to improve target association compared to wild-type Fa-G6. Analysis by CD yielded characteristic spectra of alpha helical secondary structure contents for the six non-binding alanine variants (D13A, F24A, Y25A, W28A, I31A and R32A), thus indicating that the observed loss of binding was not due to protein destabilisation (Figure S4).

### 2.4. Second-Generation Library Design and Construction

**Figure 3:**
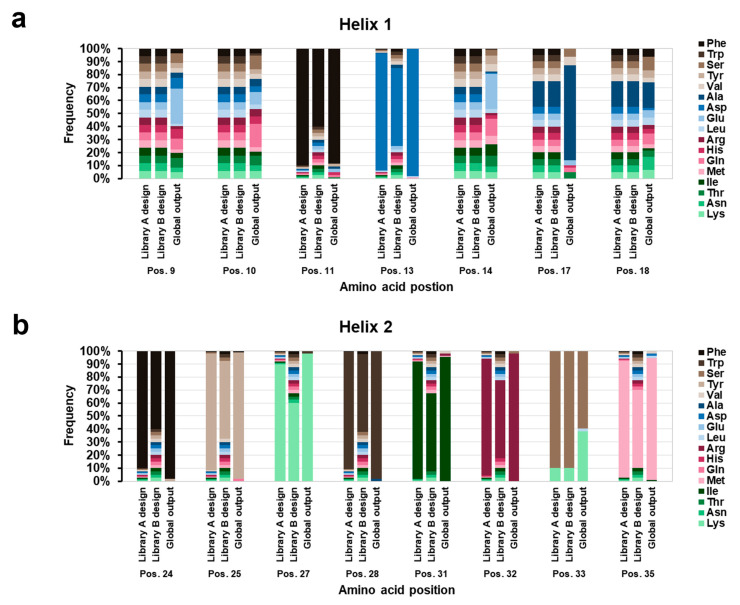
**Library design and selection output.** Amino acid distributions and frequencies of the second-generation library gene designs compared to the selection output, in the 15 randomised positions located in helices 1 (**a**) and 2 (**b**). Based on alanine scanning of the BCMA binding clone Fa-G6 two second-generation libraries, Library A and Library B, were designed. Library A was designed to be more conserved than Library B. The dataset for the distribution and frequencies of amino acids in the 15 variable positions in the output of the second-generation selections is based on sequencing data of 107 ELISA-positive unique clones (56 clones originating from Library A and 51 clones originating from Library B). Codons are represented by the three-letter abbreviation of respective amino acid, which here include all naturally occurring amino acids, except Gly, Pro, and Cys (not included in the library designs).

Two different second-generation library genes were designed based on the Fa-G6 clone. With consideration to the alanine scanning and CD analysis data, positions in Fa-G6 shown to be highly affected by alanine substitutions were more cautiously randomised than positions shown to be mildly affected. Furthermore, the two second-generation library genes were designed with the intention of generating a more conserved and a less conserved second-generation library, resulting in Library A (up to 90% conserved in specific positions) and Library B (up to 60% conserved in specific positions). In addition to the 14 positions which were randomised in the naïve library, both second-generation library genes included a 15th variable position—position 33—which allowed incorporation of either serine (90%) or lysine (10%), of which serine is the amino acid present in the naïve library gene sequence (Figure 1b). An S33K substitution in the affibody scaffold has in some cases been observed to increase the melting temperature by up to 12 °C [23]. The distribution of codons in the variable positions in the two second-generation library genes is shown in Figure 3. Codons corresponding to Gly, Pro, and Cys were excluded in the design due to potential helix disruption and dimer formation.

The two library gene mixes were cloned into two different phagemid vectors and a small amount of prepared phagemid DNA was transformed into *E. coli* for sequencing (96 colonies for each library gene). The observed amino acid distributions were in accordance with the intended designs.

### 2.5. Isolation of Second-Generation Anti-BCMA Affibody Binders

Prepared phage stocks from the second-generation libraries were used for three selection rounds against biotinylated recombinant human BCMA-rFc, in three selection tracks using Library A and six selection tracks using Library B. The successive selection cycles and nine selection tracks differed in target antigen concentration (10–140 nM), selection incubation time, total washing time, solid-phase or liquid-phase selection strategy (immobilisation of target antigen prior to incubation with phage, or incubation of target antigen with phage in solution before immobilisation, respectively), including or not including an off-rate selection strategy in cycles 2–3 (10× molar excess of non-biotinylated target antigen during the selection incubation and 100 nM non-biotinylated target antigen in the wash solution), and in elution strategy (low pH or trypsin cleavage). Following the final selection cycle, a total of 188 randomly picked clones from the final selection outputs, equally representing the nine selection tracks, were screened for binding to recombinant human BCMA-rFc and relevant controls by monoclonal phage ELISA. Sequencing analysis of 120 ELISA-positive clones revealed 107 unique second-generation candidate BCMA binders, ranging between 1–5 in frequency of appearance in the sequencing data.

A global analysis of the distribution of amino acids in the 15 variable positions of the 107 s-generation clones revealed that the output overall was more similar to the conservative design of Library A than the less conservative Library B (Figure 3). Interestingly, the amino acid occupancies in positions 13, 24, 25, 27, 28, 31, 32 and 35 were all the same as in Fa-G6 for 87% of the clones. The diversified positions 9 and 14 were both still relatively diversified in the final output, although a certain convergence to Glu in position 9 and Gln in position 14 could be observed. Position 18 also remained relatively diversified, with a distribution similar to the designed randomisation. Position 17, on the other hand, showed a striking convergence to Ala. This phenomenon agrees with the previous observation where the Fa-G6 alanine substitution variant S17A demonstrated a slightly improved binding to BCMA compared to Fa-G6 wild-type (Figure 2b). An additional interesting observation was that position 33 increased in Lys occupancy from the library design of 10% to approximately 40% in the selection output, indicating an advantage of a Lys in this position.

### 2.6. Characterisation of Second-Generation Anti-BCMA Affibodies

**Figure 4:**
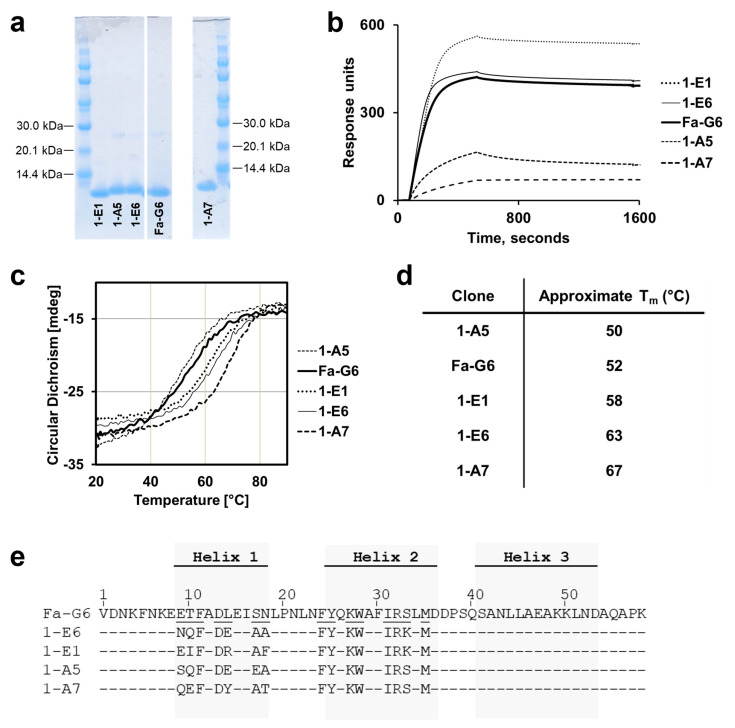
**Candidate clones from the second-generation selection campaign output, compared to the parental Fa-G6 clone.** (**a**) SDS-PAGE gels of *Escherichia coli* (*E. coli*) produced monomeric affibodies. (**b**) Overlay of single concentration (100 nM) SPR sensorgrams of clones binding to immobilised human BCMA-rFc. (**c**) Overlay of thermal denaturation profiles recorded at 221 nm. (**d**) Approximate melting temperatures (Tm) of respective clone (0.2 mg/mL), based on thermal denaturing from 20 °C to 90 °C (5 °C/min) measured at 221 nm. (**e**) Sequence alignment of the four candidate second-generation clones, compared to the parental Fa-G6 clone, showing the amino acid distribution in the 15 variable positions (underlined in the Fa-G6 sequence). The numbers above the alignment indicate the amino acid residue numbering of the affibody 58-amino-acid scaffold sequence.

From the initial set of 107 ELISA-positive and unique clones isolated from the second-generation selection campaign, a first subset of 18 clones were chosen for post-selection screening based on how many times they occurred in the sequencing data after the selection and how well they represented different sequence clusters. The 18 clones were produced as soluble, monomeric His_6_-tagged proteins for assessment of binding to BCMA by SPR and for analysis of their structural content and thermal stability using CD. Figure 4 shows the resulting data for four notable second-generation clones, denoted 1-E6, 1-E1, 1-A5 and 1-A7, which sufficiently represent the overall observed characteristics of the 18 s-generation clones, as measured by SPR and CD. The four clones could be produced with reasonable yields and purities (Figure 4a). Binding to BCMA was assessed by SPR, by injecting the proteins at 100 nM over immobilised human BCMA-rFc (Figure 4b). Both clones 1-E1 and 1-E6 showcased high response levels and slow dissociation kinetics in parity with Fa-G6 but were slightly faster in their association to BCMA. In comparison, clones 1-A5 and 1-A7 both showed considerably lower response levels, although clone 1-A7 demonstrated very slow dissociation kinetics.

CD analyses showed alpha helical secondary structure contents for the four second-generation clones (Figure S5). Thermal denaturation profiles monitored at 221 nm showed improved Tm values for three of the four clones. As much as a 15 °C increase compared to Fa-G6 was observed for clone 1-A7, with a Tm of approximately 67 °C compared to 52 °C for Fa-G6 (Figure 4c,d). Despite the high Tm, however, clone 1-A7 demonstrated inability to completely refold after thermal denaturation (Figure S5).

Comparing the amino acid sequences with Fa-G6 (Figure 4e), all four clones showed identical amino acid occupancies in helix 2 as in Fa-G6, in all but one of the eight randomised positions in helix 2. Thus, although variation at these positions was allowed in the library design, a strong selection for the original amino acid found in helix 2 of the parental Fa-G6 clone was observed. The only difference observed in helix 2 was in position 33, occupied by Lys in clones 1-E6 and 1-E1. In comparison, the parental clone Fa-G6 and second-generation clones 1-A5 and 1-A7 all had a Ser in this position. A higher degree of amino acid replacement was observed in the seven randomised positions in helix 1 (Figure 3a). The two second generation variants 1-E1 and 1-E6 both contained five amino acid changes in helix 1. These included a S17A substitution, which in the alanine scanning experiment was observed to increase the complex formation rate, and both 1-E1 and 1-E6 showed faster association kinetics than Fa-G6 in the SPR experiment. In all four clones, the original occupancies of Phe in position 11 and Asp in position 13 were retained.

Thus, the analysis of the four second-generation clones allowed us to identify two clones, 1-E1 and 1-E6, both with slightly improved association rates and with significantly improved thermal stabilities compared to Fa-G6. During the performed analyses, the 1-E1 clone showed some tendencies of aggregation upon storage, which led to the choice of clone 1-E6 as the second-generation variant to move forward with for the continued studies.

### 2.7. Binding Kinetics of Variant 1-E6

**Figure 5:**
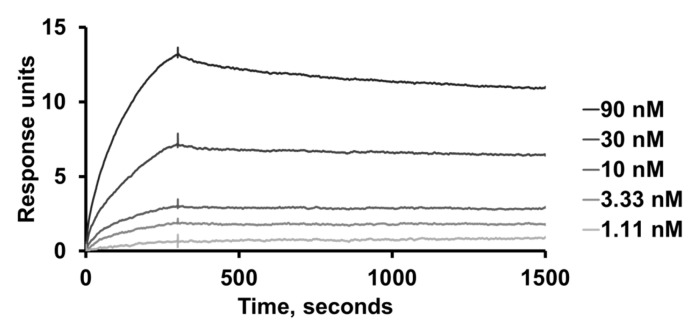
**Kinetic analysis of the BCMA-binding affibody clone 1-E6.** One representative serial dilution (1.1–90 nM) of the second-generation clone 1-E6, injected in duplicate over immobilised human BCMA-rFc. Kinetic constants *K*_D_ (dissociation equilibrium constant), *k*_a_ (association rate constant) and *k*_d_ (dissociation rate constant) were estimated from the resulting sensorgrams using BIAevaluation software (Cytiva, Uppsala, Sweden) and assuming 1:1 binding.

The binding kinetics of the second-generation clone 1-E6 was further analysed by SPR, by injecting duplicate serial dilutions over immobilised BCMA. One representative serial dilution is presented in Figure 5. The resulting sensorgrams were used to estimate the kinetic constants *K*_D_ (dissociation equilibrium constant), *k*_a_ (association rate constant) and *k*_d_ (dissociation rate constant), assuming 1:1 binding. The average values were *K*_D_ 1.4 ± 0.2 nM, *k*_a_ 9.7 × 10^4^ M^−1^s^−1^, and *k*_d_ 1.3 × 10^−4^ s^−1^.

### 2.8. Epitope Binning Studies Using APRIL and Belantamab

**Figure 6:**
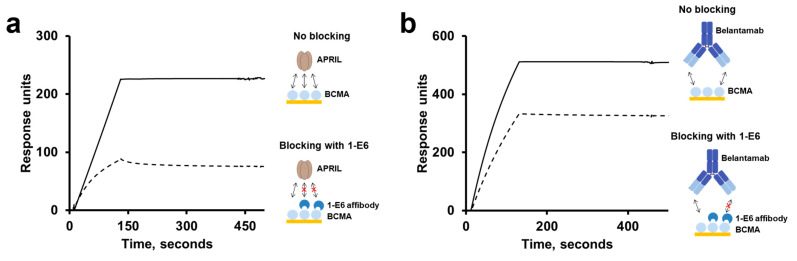
**Epitope binning studies through SPR-based blocking assay.** The response signal of BCMA-binding analyte APRIL (**a**) or belantamab (**b**) with no prior blocking (binding interaction is denoted by double-headed arrows) was compared to blocking (blocking is denoted by a red cross) with 1-E6 affibody. In the non-blocking response, running buffer was injected over the surface containing immobilised human BCMA-rFc, followed BCMA-binding analyte, 100 nM APRIL or 25 nM belantamab. Assessment of potential blocking by 1-E6 was done by first injecting 1 µM 1-E6, followed by either BCMA-binding analyte (sample run) or running buffer (reference run). The plotted blocking response corresponds to the response obtained after subtracting the reference run from the sample run.

To further characterise the binding of the 1-E6 affibody to BCMA, its potential ability to block one of the natural ligands of BCMA—APRIL—was investigated in an SPR-based blocking assay. 1-E6 was injected at a concentration of 1 µM over immobilised human BCMA-rFc, followed by an injection of 100 nM of APRIL. In a reference run, APRIL was injected at 100 nM without prior injection of 1-E6. The results showed that a lower binding response was obtained from the APRIL injection if the 1-E6 affibodies had first been injected (Figure 6a and Figure S6a). This suggests that APRIL and the 1-E6 affibody compete sterically at overlapping epitopes on BCMA. This is not surprising given the small size of the BCMA ECD.

Similarly, we also investigated if the 1-E6 affibody and anti-BCMA antibody belantamab bind to overlapping or spatially separated epitopes on BCMA. Belantamab is a humanised BCMA-binding antibody, which has been clinically evaluated with the cytotoxic payload MMAF (monomethyl auristatin F) as an ADC for MM therapy (belantamab mafodotin, Blenrep) [24]. In an SPR-based blocking assay, 1-E6 was injected at a high concentration of 10 µM over immobilised human BCMA-rFc, followed by an injection of belantamab at 25 nM. In a reference run, belantamab was injected at 25 nM without prior injection of 1-E6. A preceding injection of the 1-E6 affibody resulted in a reduced belantamab binding response, thus suggesting overlapping epitopes on BCMA for 1-E6 and belantamab as well (Figure 6b and Figure S6b).

### 2.9. Cross-Reactivity Studies

The ECD of human BCMA exhibits high degrees of sequence identity with BCMA homologues from other species, including mouse (67%), rhesus macaque (89%), and marmoset (91%). This prompted us to investigate potential cross-reactivities of the 1-E6 affibody to BCMA from these species using SPR. Sensor chip surfaces with immobilised BCMA variants were prepared, and 1-E6 was injected. To verify that the BCMA variants were functionally intact after immobilisation, human APRIL (hAPRIL) was also injected over the same sensor chip surfaces as a positive control. While strong binding of hAPRIL to all three BCMA homologues could be observed, no binding was observed for 1-E6 (Figure S7a).

Further, the observed capability of 1-E6 to interfere with the binding of APRIL to BCMA led us also to investigate if 1-E6 is cross-reactive to human TACI, a different APRIL-interacting member of the TNF receptor superfamily [25]. Here, solutions of 1-E6 and hAPRIL were injected over sensor chip surfaces containing immobilised human BCMA (BCMA-rFc) or human TACI, respectively. Both analytes showed binding to immobilised BCMA, but only hAPRIL bound to immobilised TACI (Figure S7b).

### 2.10. Cell-Binding Studies

To assess if the second-generation 1-E6 affibody could bind endogenous BCMA expressed on human MM cells, both flow cytometry and fluorescence microscopy were employed. First, 1-E6 was produced as a head-to-tail dimer with a C-terminal His_6_ tag (1-E6-1-E6-His_6_) and labelled with an AlexaFluor647 (AF647) fluorescent dye. The degree of labelling (DOL) was determined, using MALDI mass spectrometry analysis, to range between 0–3 fluorophore labels per affibody construct (Figure S8a). Labelled 1-E6-1-E6-His_6_ appeared as a somewhat smeared band and migrating slightly larger in size than unlabelled construct in an SDS-PAGE gel, likely relating to its increased size and the DOL range (Figure S8b). SPR analysis confirmed that labelled 1-E6-1-E6-His_6_ did not appear affected in its binding to human BCMA and the response signal obtained was comparable to that obtained for the unlabelled construct (Figure S8c).

Binding of the 1-E6 affibody to endogenous BCMA was analysed on the BCMA-expressing cell line MM.1s (BCMA^+^/HER2^−^) and the non-expressing control cell line SKBR3 (BCMA^−^/HER2^+^). Fluorescently labelled 1-E6-1-E6-His_6_ (200 nM) was incubated with cultivated cells, followed by analysis in flow cytometry. An AF647-labelled anti-HER2/CD16a affibody fusion (DOL range 0–3) was included as an affibody control. Phycoerythrin (PE)-labelled anti-BCMA monoclonal and polyclonal antibodies were included as positive controls, and PE-labelled isotype antibodies were included as background controls for these.

**Figure 7:**
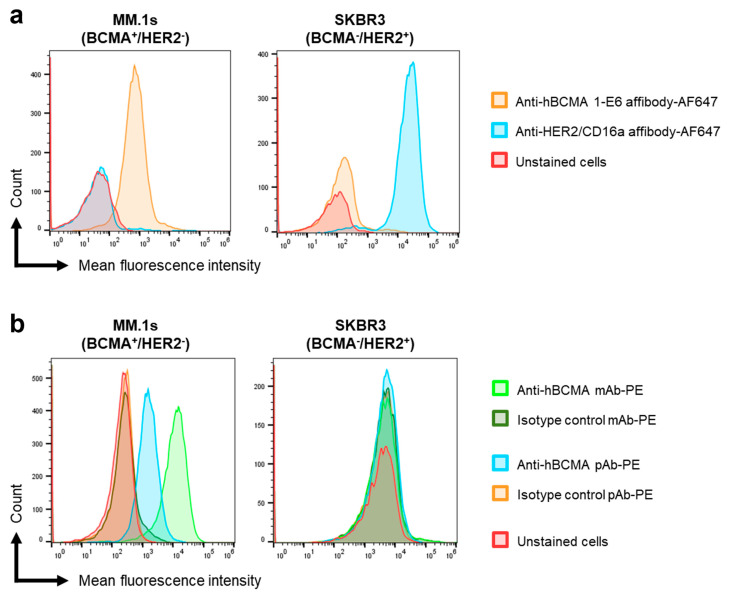
**Binding activity of anti-BCMA 1-E6 affibody to BCMA^+^ and BCMA^−^ cell lines.** Flow cytometry staining of MM.1s (BCMA^+^/HER2^−^) cells and SKBR3 (BCMA^−^/HER2^+^) cells with AlexaFluor647 (AF647)-labelled anti-BCMA 1-E6 affibody (1-E6-1-E6-His_6_) or anti-HER2 affibody control (**a**), or PE-labelled anti-BCMA antibodies (monoclonal antibody (mAb) or polyclonal antibody (pAb) or isotype control antibodies (**b**). Unstained cells were used to set the negative population.

As shown in Figure 7a, a clear shift was observed in the fluorescent signal for the BCMA^+^/HER2^−^ MM.1s cells incubated with 1-E6 affibody, but no detectable binding could be observed to BCMA^−^/HER2^+^ SKBR3 control cells. Reciprocally, the anti-HER2/CD16a affibody fusion control demonstrated no binding to the BCMA^+^/HER2^−^ MM.1s cells but bound to BCMA^−^/HER2^+^ SKBR3 cells, as expected. Although the included anti-BCMA monoclonal and polyclonal antibodies were labelled with a different fluorophore and the magnitudes of the shifts thus cannot be directly compared, the clear shifts in fluorescence signal observed for the anti-BCMA antibodies with MM.1s and negligible shifts with SKBR3 (Figure 7b) confirm that BCMA is expressed on the MM.1s cells, and not on the SKBR3 cells. Consequently, the BCMA-specificity of the 1-E6 affibody is also confirmed, supporting its applicability as a BCMA detection reagent in flow cytometry.

**Figure 8:**
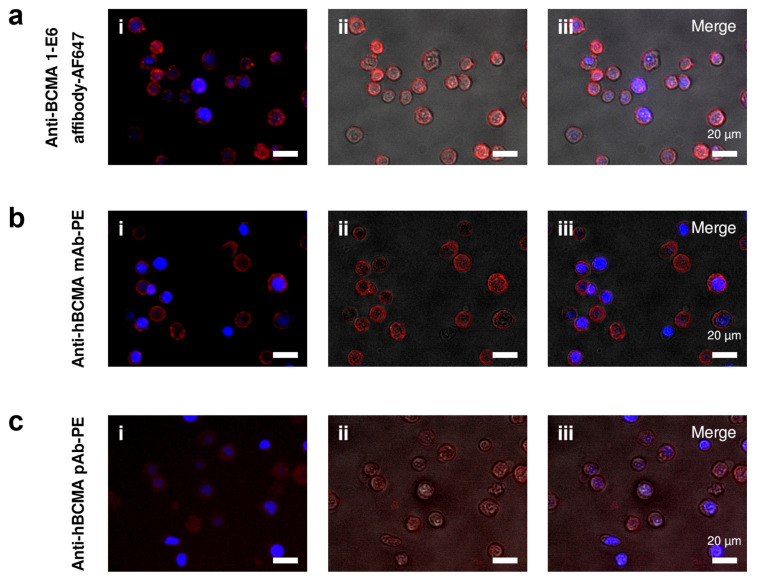
**Fluorescence and brightfield microscopy of MM.1s cells stained with anti-BCMA 1-E6 affibody.** AF647-labelled 1-E6 affibody (1-E6-1-E6-His_6_) (red) (**a**) demonstrated clear binding to BCMA-positive cell line MM.1s (BCMA^+^/HER2^−^). PE-labelled anti-BCMA mAb (red) (**b**) and pAb (red) (**c**) also demonstrated binding to the MM.1s cells. In (**a.ii**–**c.ii**)**,** the fluorescence signal of each reagent is overlapped with an image acquired with brightfield microscopy, to visualise the shape of the cells. A merge of (**i**,**ii**) is shown in (**a.iii**–**c.iii**). Nuclei are stained with DAPI (blue). Scale bars: 20 µm.

The binding specificity of the 1-E6 affibody was also analysed through fluorescence microscopy, using the same cell lines and control reagents as in the flow cytometry experiment. Here, 1 µM of fluorescently labelled 1-E6-1-E6-His_6_ was incubated with cultivated cells and then imaged by microscopy. Fluorescence-labelled 1-E6 affibody demonstrated clear cell binding to BCMA^+^/HER2^−^ MM.1s cells (Figure 8a), while no binding could be observed for the anti-HER2/CD16a affibody control (Figure S9a). Both the monoclonal and polyclonal anti-BCMA antibodies showed cell binding to MM.1s, as expected (Figure 8b,c and Figure S9b,c). In contrast, the anti-HER2/CD16a affibody control incubated with BCMA^−^/HER2^+^ SKBR3 cells demonstrated clear binding to SKBR3 cells, while none of the anti-BCMA reagents—affibody or antibody—showed any binding to this cell line (Figure S10).

Taken together, the results from the cell binding experiments validated that the fluorescence-labelled 1-E6-1-E6-His_6_ reagent was able to bind to cell surface-expressed BCMA on an MM cell line, with high specificity.

## 3. Discussion

We describe the identification of novel affibody affinity proteins that bind BCMA, an important target for MM therapies. The BCMA ECD target protein used in this study is relatively small: 45 amino acid residues, i.e., smaller than the 58-residue affibody protein. It was therefore not obvious that it should contain any surfaces amenable for interaction with the affibody binding surface, which is a relatively flat surface and lacks protein loops. However, no less than 25 unique binders could be identified after screening 144 randomly picked clones from the fourth selection cycle output.

The identification of the top candidate 1-E6 affibody variant involved two consecutive library selection steps, including the construction of two second-generation libraries. The design of these was based on binding and secondary structure content data from a set of alanine mutants of a chosen first-generation variant, Fa-G6, showing the most preferable off-rate kinetics. While the assumption was that variants with significantly higher affinities would be possible to select from these second-generation libraries, the most prominent improvement in the output clones was instead seen in their structural stabilities, measured as higher Tm values. Intriguingly, the experimental set-up of the selections did not include any explicit measures where a higher thermostability would be favoured. Rather, gradually lower antigen concentrations and increasingly extensive washings were used and expected to promote high-affinity binding.

Although the alanine scanning experiment of the parental clone Fa-G6 showed that alanine substitutions at all seven positions in helix 2 severely affected binding, it is somewhat surprising that the output clones from the second-generation libraries almost exclusively contained the original Fa-G6 amino acid at six of these seven re-randomised positions, and that no replacement to chemically similar amino acids were observed. The output also showed a striking convergence to alanine in position 17. In the alanine scanning experiment of the Fa-G6 variant, it was observed that the S17A mutant showcased a high-affinity BCMA binding profile, with a faster complex formation rate than Fa-G6 wild-type, suggesting that the selection conditions to at least some extent promoted clones with improved binding.

Position 33 is not located in the binding surface of the affibody scaffold, but it is surface exposed and has been implied to be important for the scaffold stability [23]. In both second-generation libraries this position, occupied by serine in the parental clone Fa-G6, was conservatively included in the randomisation and designed to be occupied by 90% serine and 10% lysine. However, the global analysis of the output demonstrated an increase of lysine to 40% occupancy, suggesting a favourable contribution during selections. CD spectroscopy showed that the second-generation clones 1-E1 and 1-E6, both containing lysine in this position, had improved Tm values by approximately 6 and 11 °C, respectively, supporting the notion of its importance for the structural stability. However, it is worth noting that the second-generation clone 1-A7, containing a serine at position 33, exhibited the most improved Tm, with a 15 °C increase compared to the parental Fa-G6 clone. However, this large increase in stability was not associated with a stronger binding affinity to BCMA.

No binding was observed for the 1-E6 affibody to BCMA homologues from mouse, rhesus and marmoset, despite their high degrees of sequence identity to human BCMA in their ECDs. However, even if human and marmoset BCMA share 91% sequence identity, five amino acid positions and a small gap differ between the two proteins that could explain the findings. We found that the 1-E6 affibody was able to compete for binding to BCMA with APRIL, a native ligand to BCMA involved in the promotion of MM cell proliferation and survival [18]. Similarly, the anti-BCMA monoclonal antibody belantamab has also been shown to be able to compete with APRIL for BCMA binding. This competition has been suggested to be one of belantamab’s mode of actions, when clinically investigated as an antibody-drug conjugate [26]. Our data indicates that the respective epitopes of the 1-E6 affibody and belantamab are overlapping, or partially overlapping, and are coinciding with the binding site of APRIL on BCMA. This finding may be expected considering the small size of the BCMA ECD. It is appealing to theorise that the shared epitope space of the BCMA-binders APRIL, belantamab and the 1-E6 affibody may be a so-called “epitope hotspot” of the BCMA ECD. As suggested for belantamab, this may indicate that the 1-E6 affibody may inhibit BCMA-induced proliferation, via blocking of APRIL, as its therapeutic mode of action. On the other hand, it could also suggest that its therapeutic efficacy may be negatively affected by competition with soluble APRIL.

Resistance-driving mutations in BCMA have been described to negatively affect BCMA-binding reagents [27]. However, in the same study APRIL was shown to be able to bind BCMA containing these resistance-driving mutations. Thus, since 1-E6 demonstrated overlapping binding epitopes with APRIL on BCMA, it is not impossible that 1-E6 may also recognise such BCMA variants.

An interesting observation was that two of the initially isolated binders with the highest binding responses (Fa-B3 and Ft-H11) each contained a single cysteine amino acid, and that substituting these cysteines for serines led to the loss of binding. In fact, a similar observation has earlier been described in the selection of affibodies against the Alzheimer’s disease-related amyloid β (Aβ) 1–40 peptide. Also here, homodimer formation of affibody monomers via a disulfide bridge between internal cysteines was found to be essential for efficient target binding. The Aβ binding of two cysteine-to-serine mutants was almost completely restored by homodimerisation through tandem head-to-tail fusion [28]. Subsequent structural studies by NMR of the complex between a disulfide-bridged affibody homodimer and the Aβ 1–40 peptide showed a cooperative binding mode, involving each affibody unit interacting with a distinct epitope on a single Aβ 1–40 peptide monomer, providing an explanation for the observations [29]. It is tempting to speculate that the cysteine-containing affibody variants selected against the small BCMA target in this work form similar affibody-target complexes of 2:1 stoichiometry. One advantage of the affibody scaffold is that it does not contain any native cysteine, thus allowing for introduction of cysteine in the coding sequence post-selection, to provide opportunities for site-directed conjugation of various functional groups, including fluorophores, biotin, cytotoxic drugs and chelators. Thus, affibody variants that contain additional cysteines of importance for homodimerisation and target binding are less attractive for many biotechnological or medical applications. However, as shown for the Aβ 1–40 peptide-binding affibodies, tandem head-to-tail fusion of cysteine-to-serine mutants could still be of interest, but this was not evaluated for the cysteine-containing anti-BCMA affibodies in this study.

The second-generation 1-E6 affibody (1-E6-1-E6-His_6_) clearly demonstrated specific binding to BCMA expressed on MM.1s cells in flow cytometry and in fluorescence microscopy. Although not directly comparable due to the different fluorochromes used, the binding activity was analogous to that of BCMA-binding antibodies. In addition, no binding was observed for BCMA^−^ SKBR3 cells. In these assays, it is inconclusive if any internalisation has occurred. If the 1-E6 homodimer induces BCMA internalisation it could affect its usage in therapeutic constructs, depending on the intended mode of action, e.g., internalisation could be beneficial for its use in drug conjugates, but negatively affect its use for immune cell activation.

The 1-E6 homodimer preparation used was randomly labelled using primary amines present in the protein sequence, meaning that the prepared sample was of a heterogeneous nature and that the binding to BCMA could have been affected. This was especially a risk considering the lysine residue in position 27, which was observed to be essential for binding in the alanine scanning. We did, however, not observe any diminished BCMA binding by labelled 1-E6 homodimer in SPR, but future studies with labelled protein could potentially be done with affibodies labelled in a site-specific manner as mentioned above, e.g., via introduction of a unique C-terminal cysteine, for coupling via maleimide chemistry. Nevertheless, our results show the feasibility of using a BCMA-binding affibody in both flow cytometry and microscopy assay settings, for the detection of BCMA-expressing cells. It should be noted that since affibodies are devoid of any Fc regions, the use of so-called Fc blockers, commonly used to compete out unwanted interactions between diagnostic antibodies and cell surface Fc receptors, are not needed.

A BCMA-directed CAR T cell therapy, anitocabtagene autoleucel (anito-cel; CART-ddBCMA), has been assessed in a Phase I clinical trial (NCT04155749) and shown an impressive 100% overall response rate (ORR) in heavily pretreated relapsed or refractory MM (RRMM) patients [30]. The CAR component constitutes an 8 kDa BCMA-targeting D-domain, based on a *de novo*-designed three-helix bundle scaffold. The D-domain scaffold does not share sequence similarity with affibodies, but both scaffolds appear similar in their overall topology [31,32]. Although its functional success may be due to several different factors, it cannot be ruled out that the structural characteristics of the CAR component plays an important role, including its small size and compact structure, which consequently allow for a higher CAR expression level during manufacturing and improved functional efficacy compared to a scFv CAR [31]. Thus, the encouraging clinical data presented for anito-cel provides a compelling motivation for the further development and use of alternative, non-immunoglobulin scaffolds, including affibodies, in therapeutic applications. In fact, an initial study on the use of the anti-BCMA 1-E6 affibody—developed in the present work—as the tumour antigen-binding unit in a BCMA × CD16a dual engager for NK cell-mediated killing of MM.1s cells in vitro, has yielded encouraging results [22].

Anti-drug antibodies (ADAs) may be elicited against proteins drugs, including humanised or even fully human monoclonal antibodies [33]. Such antibodies may affect both safety and efficacy. The non-human origin of the affibody scaffold may therefore raise immunogenicity concerns. In two clinical studies (52 weeks and up to three years, respectively) of the affibody-based drug Izokibep (an IL-17A inhibitor), ADAs were detected in some individuals. However, these responses were reported not to affect pharmacokinetic (PK) parameters, safety, or efficacy [34,35]. This holds promise that the described anti-BCMA affibody could be included as the targeting unit in drugs aimed for MM therapy in humans.

In summary, this work demonstrates the successful isolation and development of anti-BCMA affibodies, using phage display technology. Characterisation of the second-generation anti-BCMA clone 1-E6 demonstrated its high thermostability and specific, high-affinity binding to BCMA, highlighting the potential for broad applicability of this small, non-immunoglobulin reagent in e.g., diagnostic and medical applications.

## 4. Materials and Methods

### 4.1. First-Generation Phage Library and Stock Preparation

Preparation of an M13 phage library of affibodies and the subsequent production of a naïve affibody-displaying phage stock based on the pAffi-1 phagemid [28] were done as described in detail in [22]. The corresponding phage library preparation and phage stock production using the pAffi-100 phagemid were performed similarly. pAffi-100 differs from the pAffi-1 phagemid in that it contains a trypsin protease cleavage site before the amber stop codon, which is followed by full-length M13 phage coat protein III.

### 4.2. Selection of First-Generation Binders to BCMA Using Phage Display

Four selection cycles were performed in three parallel tracks, with different selection conditions for each track. Biotinylated human BCMA-rFc (human TNFRSF17/BCMA/CD269 Protein (His & human IgG1 Fc tag, Biotinylated), cat. no. 10620-H03H-B, Sino Biological, Eschborn, Germany), corresponding to residues 1–54 of Uniprot entry Q02223, was used as the target antigen. Biotinylated human BCMA-rFc (150 nM (cycle 1), 100 nM (cycle 2) and 50 nM (cycles 3–4)) was immobilised on 1.5 mg streptavidin (SA)-coated paramagnetic beads (Dynabeads M-280 Streptavidin, cat. no. 11205D, Invitrogen, Waltham, MA, USA) for 1 h at room temperature (RT) and under constant end-over-end (eoe) rotation. Affibody-displaying phage stocks generated from the libraries based on pAffi-1 and pAffi-100 phagemids were added to the immobilised biotinylated human BCMA-rFc (pre-blocked with 1% (*w*/*v*) bovine serum albumin (BSA) in PBS-T (PBS (150 mM NaCl, 8 mM Na_2_HPO4, 2 mM NaH_2_PO_4_) supplemented with 0.05% (*v*/*v*) Tween-20, pH 7.4)) for selection incubation for 3 h (cycle 1), 4 h (cycle 2), 2 h (cycle 3) or 1 h and 20 min (cycle 4), at RT with eoe rotation. Prior to the selection incubation step in cycles 2–4, negative selection was performed to remove phage carrying binders against SA and Fc, by pre-incubating the phage stocks in PBS-T with 0.1% (*w*/*v*) BSA and SA-beads containing biotinylated trastuzumab mAb (contains Fc region) (Herceptin, Roche, Basel, Germany) for 1 h at RT with eoe rotation.

After several washes with PBS-T (eoe at RT), bound phage was eluted with 0.3 M acetic acid, pH 2.8, for 15 min eoe at RT, followed by neutralisation with an equal volume of 1 M Tris-HCl, pH 8; or eluted with 0.25 mg/mL trypsin (Gibco Life Technologies, Carlsbad, CA, USA) for 30 min, eoe at RT. After selection cycles 1–3, helper phage M13K07 for pAffi-1 or KM13 for pAffi-100 were used to amplify new phage stocks in *E. coli* XL-1 Blue cells (Agilent, Santa Clara, CA, USA), for use as the input phage in the subsequent selection cycle. Phage stock titres were measured by infecting *E. coli* XL-1 Blue and performing spot titration on carbenicillin plates (30 g/L blood agar, 100 µg/mL carbenicillin). Colony PCR was used to analyse the percentage of infected colonies carrying phagemids with the correct affibody insert size.

### 4.3. Surface Plasmon Resonance Characterisation of First-Generation Candidate BCMA-Binding Clones

ELISA-positive and unique clones (Supplementary Materials and Methods) were subcloned for soluble expression in *E. coli* as fusion proteins with an N-terminal His_6_ tag and a C-terminal ABD (His_6_-affibody-ABD format). DNA fragments encoding the clones of interest were first amplified by PCR from the library phagemids with specific primers introducing restriction recognition sites for Xhol and AscI restriction enzymes. The fragments were digested with XhoI and AscI and then ligated to a T7 promoter-based *E. coli* expression vector digested with the same restriction enzymes. The resulting DNA constructs were sequence verified using Sanger sequencing (Microsynth) and transformed into *E. coli* BL21(DE3) for expression. Cells were chemically lysed, and His_6_-tagged fusion proteins were purified under denaturing conditions using HisPur Cobalt IMAC Resin (cat. no. 89966, Thermo Scientific, Waltham, MA, USA) for IMAC purification. Following purification, the protein buffer was exchanged to PBS using PD-10 desalting columns (cat. no. 17085101, Cytiva, Uppsala, Sweden). Concentrations were estimated based on absorbances measured at 280 nm and using molecular weights (MW) and extinction coefficients calculated from the amino acid sequences. SDS-PAGE analysis under reducing conditions was performed to confirm the purities and concentrations.

A Biacore T200 instrument (Cytiva, Uppsala, Sweden) was used to analyse the real-time interactions of the fusion proteins with BCMA, by SPR. The protein ligand human BCMA-rFc (cat. no. 10620-H15H, Sino Biological, Eschborn, Germany) was immobilised on a Series S CM5 sensor chip (Cytiva, Uppsala, Sweden) by amine coupling, using the manufacturer’s instructions. One flow cell was activated and deactivated to be used as a reference cell. The affibody fusion proteins were injected at 200 nM over the flow cells. After each run cycle, the flow cells were regenerated with 10 mM HCl. PBS-T was used as the running buffer and sample buffer.

### 4.4. Alanine Scanning of the First-Generation BCMA-Binding Clone Fa-G6

Fa-G6 was subcloned into a T7 promoter-based *E. coli* expression vector for soluble expression of proteins with an N-terminal His_6_ tag (His_6_-affibody format), similarly as described above. The expression plasmid containing the Fa-G6 construct was subsequently used as the DNA template for alanine scanning mutagenesis. A total of 14 individual mutagenesis PCR reactions were performed to substitute the original codons in the 14 variable positions (residues 9, 10, 11, 13, 14, 17, 18, 24, 25, 27, 28, 31, 32, and 35) to alanine codons, using primers designed for each position. The 14 alanine substitution variants were sequence verified using Sanger sequencing (Microsynth, Göttingen, Germany). *E. coli* BL21(DE3) cells were separately transformed with individual plasmids encoding the N-terminally His_6_-tagged alanine variants, as well as the Fa-G6 wild-type construct. Production, purification under denaturing conditions, buffer exchange, absorbance measurement, and SDS-PAGE analysis were performed as described above.

A Biacore T200 instrument (Cytiva, Uppsala, Sweden) was used to analyse the interactions of the Fa-G6 wild-type and the 14 alanine variants with BCMA in real time. Immobilisation of the protein ligand human BCMA-rFc (cat. no. 10620-H15H, Sino Biological, Eschborn, Germany), generation of a reference cell, binding analysis and regeneration of flow cells were performed as described above. PBS-T was used as the running buffer and sample buffer.

### 4.5. Second-Generation Library Construction, Selection and Monoclonal Phage ELISA

Two M13 phage display second-generation library (Library A and Library B) genes were designed, based the Fa-G6 clone, and synthesised as oligonucleotides (121 bases) encoding amino acids 3–41 (reverse complementary strand) of the Z domain [36]. The oligonucleotides were synthesised by ELLA Biotech (Furstenfeldbruck, Germany) using custom mixtures of trinucleotide codon building blocks in the 14 variable positions (residues 9, 10, 11, 13, 14, 17, 18, 24, 25, 27, 28, 31, 32 and 35). Validation of the library gene designs was done by sequencing (Microsynth, Göttingen, Germany) of 96 individually obtained colonies following a small-scale test ligation: Library A and Library B oligonucleotides were PCR-amplified using specific primers to introduce restriction recognition sites for XhoI and NheI restriction enzymes, then XhoI/NheI digested and ligated to XhoI/NheI digested pAffi-1 (Library A) or pAffi-100 (Library B), and finally transformed into electrocompetent ER2738 *E. coli* cells (F’, *glnV* amber suppressor) (Lucigen, San Antonio, TX, USA) by electroporation. Following library validation, full-scale libraries were generated as described above, using ligation reaction mixes of 5.4 µg of XhoI/NheI-digested PCR products of Library A or Library B with 30 µg of XhoI/NheI-digested pAffi-1 or pAffi-100, respectively. Recovered post-electroporation transformants were analysed by titration via spreading of dilution series on ampicillin plates (30 g/L blood agar, 100 µg/mL ampicillin) and cultivated overnight in TSB+Y supplemented with 1.5% (*w*/*v*) glucose and 100 µg/mL ampicillin. Overnight cultures were pelleted by centrifugation and resuspended in cold 40% (*v*/*v*) glycerol, followed by freezing at -80 °C. Library size (diversity) was calculated from the post-electroporation titrations, which for Library A in pAffi-1 was approximately 3.4 × 10^7^ and for Library B in pAffi-100 was approximately 5.9 × 10^7^.

Second-generation affibody displaying phage stocks based on Library A in pAffi-1 and Library B in pAffi-100 were prepared as described previously [22]. Recombinant, biotinylated human BCMA (human TNFRS/BCMA/CD269 Protein (His & Fc tag), Biotinylated, cat. no. 10620-H03H-B, Sino Biological, Eschborn, Germany), corresponding to residues 1–54 of Uniprot entry Q02223, was used as the target antigen. Three selection cycles were performed in nine parallel tracks (three using Library A and six using Library B), with different selection conditions for each track (Supplementary Materials and Methods).

Following selection cycle 3, 20–21 bacterial colonies generating the correct affibody insert size in colony PCR and equally representing the nine selection tracks were individually grown for monoclonal phage ELISA. MaxiSorp ELISA plates (Clear Flat-Bottom Immuno Nonsterile 384-Well Plates, cat. no. 464718, Thermo Fisher Scientific, Waltham, MA, USA) were coated with 1 µg/mL target antigen biotinylated human BCMA-Fc or control antigens: 20 µg/mL HSA, 10 µg/ul SA or 10 µg/mL trastuzumab (Fc control) (Herceptin, Roche, Basel, Switzerland); all in 100 mM sodium carbonate buffer, pH 9.6, for 16–18 h at 4 °C with slow shaking. The amplified monoclonal phage stocks were subjected to ELISA, as described following the naïve selection (Supplementary Materials and Methods). Candidates that were considered ELISA-positive clones had high HSA signals and relatively high signals to BCMA, compared to signals observed for SA and trastuzumab (Fc control) and the unrelated antigen control. Following ELISA screening, a total of 120 ELISA-positive clones were sent for DNA sequencing by Sanger sequencing (Microsynth).

### 4.6. Characterisation of Second-Generation Candidate BCMA-Binding Clones

A subset of 18 s-generation BCMA-binding clones, equally representing the nine selection tracks, were chosen for expression as soluble proteins with a C-terminal His_6_ tag (affibody-His_6_ format). DNA fragments encoding the 18 clones and the Fa-G6 wild-type clone were amplified with specific primers, designed to introduce overhangs complementary to ends of a linearised T7 promoter-based *E. coli* expression vector for cloning using In-Fusion HD Cloning Kit (TakaraBio, Gothenburg, Sweden). The resulting constructs were sequence-verified using Sanger sequencing (Microsynth). Production of the 18 constructs and the Fa-G6 wild-type clone, purification under denaturing conditions, buffer exchange, absorbance measurement and SDS-PAGE analysis were performed as described above.

A Biacore T200 instrument (Cytiva, Uppsala, Sweden) was used to analyse the interactions of the second-generation binding clones and the Fa-G6 wild-type clone with BCMA in real time. Immobilisation of the protein ligand human BCMA-rFc (cat. no. 10620-H15H, Sino Biological, Eschborn, Germany), generation of a reference cell, binding analysis, and regeneration of surfaces were performed as described above, but with binding clones diluted to 100 nM for binding analysis. PBS-T was used as the running buffer and sample buffer.

Secondary structure contents and thermal denaturation profiles were analysed as described for the first-generation clones (Supplementary Materials and Methods), but using samples diluted to 0.2 mg/mL.

### 4.7. Determination of Binding Kinetics Using SPR

Kinetic analysis of the second-generation clone 1-E6 was conducted using SPR and a Biacore T200 instrument (Cytiva, Uppsala, Sweden). Immobilisation of the protein ligand human BCMA-rFc (cat. no. 10620-H15H, Sino Biological, Eschborn, Germany), generation of a reference cell, and regeneration of flow cells were performed as described above, but with a lower surface density. PBS-T was used as the running buffer and sample buffer. Duplicate 1:3 serial dilutions of 1-E6 (affibody-His_6_ format) ranging 1.11–90 nM, were injected over the surfaces. Kinetic constants were estimated from the resulting sensorgrams using BIAevaluation software (Cytiva, Uppsala, Sweden) and assuming 1:1 binding.

### 4.8. Epitope Binning Analyses by SPR

For epitope binning analyses of the 1-E6 affibody, blocking assays were set up using a Biacore T200 instrument (Cytiva, Uppsala, Sweden). Immobilisation of the protein ligand human BCMA-rFc (cat. no. 10620-H15H, Sino Biological, Eschborn, Germany), generation of a reference cell, and regeneration of flow cells were performed as described above. PBS-T was used as the running buffer and sample buffer. 1 µM of 1-E6 (affibody-His_6_ format) was injected over immobilised human BCMA-rFc, followed by an injection of running buffer, 100 nM of APRIL (R&D systems, Minneapolis, MN, USA), or 25 nM belantamab (Belantamab Biosimilar—Research Grade, cat. no. ICH5011, ichorbio, Wantage, UK). Reference runs were performed where running buffer was injected, followed by either 100 nM APRIL or 25 nM belantamab.

### 4.9. Flow Cytometry Analyses of BCMA-Binding 1-E6 Homodimer

Cultured MM.1s and SKBR3 cells were detached from cultivation flasks with Versene solution (cat. no. 150400-066, Gibco, Thermo Scientific, Waltham, MA, USA). Cells were washed with PBS, and approximately 10^5^ cells were seeded per well in a 96-well plate (TC treated V-bottom, Corning, Thermo Scientific, Waltham, MA, USA). The cells were stained for viability (LIVE/DEAD Fixable Aqua Dead Cell Stain Kit, cat. no. L34957, Invitrogen, Thermo Fisher Scientific, Waltham, MA, USA) for 10 min at 4 °C in the dark. Cells were washed twice with Flow staining buffer (eBioscience™ Flow Cytometry Staining Buffer, cat. no. 00-4222-26, Invitrogen, Thermo Fisher Scientific, Waltham, MA, USA), then blocked with Fc Receptor Blocking Reagent (Human Seroblock, cat. no. BUF070B, Bio-Rad). 200 nM AF647-labelled 1-E6 affibody (1-E6-1-E6-His_6_) or control was added to the cells and incubated for 20 min at 4 °C in the dark. The controls used were AF647-labelled affibody control (Alexa Fluor 647 NHS Ester (Succinimidyl Ester), cat. no. A20006, Invitrogen, Thermo Fisher Scientific, Waltham, MA, USA) (binding unrelated targets HER2 and CD16a), PE-labelled anti-BCMA monoclonal antibody (PE anti-human CD269/BCMA Antibody, cat. no. 357503, Biolegend, San Diego, CA, USA), anti-BCMA polyclonal antibody (Human BCMA/TNFRSF17 PE-conjugated Antibody, cat. no. FAB193P, R&D Systems, Minneapolis, MN, USA) or corresponding isotype control antibodies (PE Mouse IgG2a, κ Isotype Ctrl (FC) Antibody, cat. no. 400213, Biolegend, San Diego, CA, USA; Goat IgG PE-conjugated Antibody, cat. no. IC108P, R&D Systems, Minneapolis, MN, USA). Cells were washed twice with Flow staining buffer, fixed with Fixation buffer (Flow Cytometry Fixation Buffer, cat. no. FC004, R&D Systems, Minneapolis, MN, USA) for 10 min at RT, then washed again and resuspended in Flow staining buffer for flow cytometric measurement (Sony ID7000 Spectral Cell Analyzer). Mean fluorescence intensity (MFI) was plotted and analysed using FlowJo Software (BD Life Sciences, Ashland, OR, USA).

### 4.10. Fluorescence and Brightfield Microscopy

SKBR3 cells were prepared by enzymatic detachment (Gibco™ TrypLE™ Express Enzyme (1X), phenol red, cat. no. 12605-028, Gibco, Thermo Scientific, Waltham, MA, USA), seeding at approximately 2 × 10^4^ per well a 96-well plate (Thermo Scientific™ Nunc™ MicroWell™ 96-Well, Nunclon Delta-Treated, Flat-Bottom Microplate, cat. no. 10212811, Thermo Scientific, Waltham, MA, USA), and allowing the cells to adhere and grow in the plate for 7 days. Cultured MM.1s cells were detached from cultivation flasks with Versene solution (cat. no. 150400-066, Gibco, Thermo Scientific, Waltham, MA, USA). Approximately 10^5^ cells per well were transferred to a 96-well conical bottom plate (Nunc™ 96-Well Polystyrene Conical Bottom MicroWell™ Plates, cat. no. 249935, Thermo Scientific, Waltham, MA, USA).

The cells were washed twice with PBS, then blocked with 1% (*v*/*v*) goat serum (cat. no. G9023, Sigma-Aldrich, Saint Louis, MO, USA) in PBS for 5 min at RT. 1 µM AF647-labelled 1-E6 affibody (1-E6-1-E6-His_6_) or control was added to the cells and incubated for 20 min at 4 °C in the dark. The controls were 1 µM control affibody (AF647-labelled anti-HER2/CD16a affibody), or anti-BCMA antibody (PE anti-human CD269/BCMA Antibody, cat. no. 357503, Biolegend, San Diego, CA, USA), anti-BCMA polyclonal antibody (Human BCMA/TNFRSF17 PE-conjugated Antibody, cat. no. FAB193P, R&D Systems, Minneapolis, MN, USA) or corresponding isotype control (PE Mouse IgG2a, κ Isotype Ctrl (FC) Antibody, cat. no. 400213, Biolegend, San Diego, CA, USA; Goat IgG PE-conjugated Antibody, cat. no. IC108P, R&D Systems, Minneapolis, MN, USA). Anti-BCMA antibodies and isotype controls were added at 5x the recommended reagent volume per cell according to the manufacturers. Cells were washed twice with PBS, then fixed with 4% paraformaldehyde (cat. no. 15424389, Thermo Scientific, Waltham, MA, USA) for 10 min at RT, then washed twice with PBS again. Cell nuclei were stained with DAPI (cat. no. D9542, Sigma-Aldrich, Saint Louis, MO, USA) for 10 min at RT. Cells were washed with PBS for 5 min. Lastly, the MM.1s cells were transferred to a 96-well flat-bottom plate (Thermo Scientific™ Nunc™ MicroWell™ 96-Well, Nunclon Delta-Treated, Flat-Bottom Microplate, cat. no. 10212811, Thermo Scientific, Waltham, MA, USA). Cell imaging was done using widefield microscopy (DMI6000 B microscope, Leica Microsystems, Wetzlar, Germany).

## 5. Patent

The following patent application has resulted from the work reported in this manuscript: “Novel polypeptides”, publication number WO/2023/232912 [37].

## Data Availability

The original contributions presented in this study are included in the article/Supplementary Material. Further inquiries can be directed to the corresponding author(s).

## Supplementary Materials

The following supporting information can be downloaded at: https://www.mdpi.com/article/10.3390/ijms26115186/s1.
