# Supplementary material for: An Anti-BCMA Affibody Affinity Protein for Therapeutic and Diagnostic Use in Multiple Myeloma
Source: Int J Mol Sci. 2025 May 28;26(11):5186. doi: 10.3390/ijms26115186 (PMC12154184; doi:10.3390/ijms26115186)
Supplement: Supplementary file 1 [file ijms-26-05186-s001.zip › Proofread Supplementary Information - clean version 2025-05-27.pdf]

Supplementary Information

An anti-BCMA affibody affinity protein for therapeutic and diagnostic use in multiple myeloma

Kim Anh Giang[1], Johan Nilvebrant[1], Hao Liu[1], Harpa Káradóttir[2], Yumei Diao[2], Stefan Gelius Svensson[2], Per-Åke Nygren[1,*]

1 Department of Protein Science, KTH-Royal Institute of Technology, Stockholm SE-114 28, Sweden; kagiang@kth.se (K.A.G.); johanni@kth.se (J.N.); lhhot150@gmail.com (H.L.)

2 Oncopeptides AB, Solna SE-171 48, Sweden; harpak91@gmail.com (H.K.); yumei.diao@oncopeptides.com (Y.D.); stefan.svensson.gelius@oncopeptides.com (S.S.G.)

* Correspondence: perake@kth.se (P.-Å.N.); Tel.: +46-70 834 95 53

1. Supplementary Material and Methods

1.1. Monoclonal phage ELISA screening of BCMA-binding candidates

MaxiSorp ELISA plates (Clear Flat-Bottom Immuno Nonsterile 384-Well Plates, cat. no. 464718, Thermo Fisher Scientific, Waltham, MA, USA) were coated with 5 μg/ml target antigen human BCMA-rFc (Recombinant Human BCMA Protein (ECD, rFc Tag), cat. no. 10620-H15H (corresponding to residues 1–54 of NCBI entry NP_001183.2 fused to a rabbit Fc), Sino Biological, Eschborn, Germany) or control antigens: 15 μg/ml human serum albumin (HSA; product no. SRP6182, Sigma), for assessment of proper display of the expression cassette containing a tripartite fusion protein including an affibody, an affibody albumin binding domain and the truncated protein III; 10 μg/ml trastuzumab (Herceptin, Roche, Basel, Switzerland), as a control for binding to the Fc tag; or 5 μg/ml unrelated control antigen (Human CD38, His Tag, Acro Biosystems (Newark, DE, USA), cat. no. CD8-H5224 corresponding to residues 43–300 of NCBI entry NP_001766.2); all in 100 mM sodium carbonate buffer, pH 8.5, for 16–18 h at 4 °C with slow shaking. 144 bacterial colonies obtained from the cycle 4 eluate were randomly picked and individually grown in TSB+Y/Carb (30 g/l tryptic soy broth, 5 g/l yeast extract, 100 μg/ml carbenicillin) and amplified using M13K07 helper phage. The amplified monoclonal phage stocks were incubated with target or control antigen coated on the ELISA plate, for 1 h at room temperature (RT) with slow shaking. After washing the plate three times with PBS-T (PBS (150 mM NaCl, 8 mM Na_2HPO4, 2 mM NaH_2PO_4) supplemented with 0.05 % (v/v) Tween-20, pH 7.4)), α-M13-HRP antibody (Sigma-Aldrich, Stockholm, Sweden) diluted 1:5000 in PBS-T was added. After incubation for 30 min at RT with slow shaking, the plate was washed twice with PBS-T and once with PBS. Bound α-M13-HRP antibody was detected using TMB substrate (TMB Substrate Kit, Thermo Fisher Scientific, Waltham, MA, USA) and the reactions were stopped by the addition of 2 M H2SO4 after 15–25 min. Absorbance at 450 nm was measured using a CLARIOstar microplate reader (BMG Labtech, Ortenberg, Germany). Candidates that were considered ELISA-positive clones had high HSA signals and relatively high signals to human BCMA-rFc, compared to signals observed for trastuzumab (Fc control) and the unrelated antigen control. ELISA-positive clones generating the correct affibody insert size in colony PCR were sent for DNA sequencing by Sanger sequencing (Microsynth Seqlab Sanger Sequencing Service, Microsynth, Göttingen, Germany).

1.2. Analyses of cysteine-to-serine mutation variants of first-generation BCMA-binding clones Fa-B3 and Ft-H11

The His_6-affibody-ABD expression plasmids containing the Fa-B3 and Ft-H11 genes were used as the DNA templates for cysteine-to-serine mutations. Individual mutagenesis PCR reactions were performed to substitute the original codons in residue position 32 to serine codons, generating C32S variants of Fa-B3 and Ft-H11. The C32S variants were sequence verified using Sanger sequencing (Microsynth, Göttingen, Germany). Fa-B3 C32S and Ft-H11 C32S were produced recombinantly in *E. coli* BL21(DE3), purified under denaturing conditions and buffer exchanged as described in the main text. Presence of S-S bridged homodimers was investigated by SDS-PAGE, using both reducing and non-reducing sample loading buffer. Binding to BCMA was investigated by SPR, using a Biacore T200 instrument (Cytiva, Uppsala, Sweden). The protein ligand human BCMA-rFc (cat. no. 10620-H15H, Sino Biological, Eschborn, Germany) was immobilised on a Series S CM5 sensor chip (Cytiva, Uppsala, Sweden) by amine coupling, using the manufacturer's instructions. One flow cell was activated and deactivated to be used as a reference cell. Fa-B3, Ft-H11, and the corresponding C32S mutation variants were injected at 200 nM over the flow cells. After each run cycle, the flow cells were regenerated with 10 mM HCl. PBS-T was used as the running buffer and sample buffer.

1.3. Circular dichroism of first-generation BCMA-binding clones Fa-G6, Ft-B11 and Ft-C11

DNA fragments encoding the BCMA-binding clones Fa-G6, Ft-B11 and Ft-C11 were subcloned into a T7 promoter-based *E. coli* expression vector for soluble expression of proteins with an N-terminal His_6 tag (affibody-His_6 format). Production, purification under denaturing conditions, buffer exchange, absorbance measurement and SDS-PAGE analysis were performed as described in the main text. The purified proteins were diluted to 0.5 mg/ml in PBS and analysed in a Chirascan CD Spectrometer (Applied Photophysics, Leatherhead, United Kingdom) and using optical path length 1 mm. Secondary structure contents were measured at 195–260 nm at 20 °C (average of 5 scans). Melting temperatures were calculated from the thermal denaturation profiles, obtained by recording the ellipticity at 221 nm during heating from 20 to 90°C (5 °C/min). Refolding capability was determined by measuring the secondary structure content as above, after the heated protein samples had cooled down to 20 °C.

1.4. Selection of second-generation binders to BCMA using phage display

Three selection cycles were performed in nine parallel tracks, with different selection conditions for each track. Each selection strategy below describes three tracks—one track using Library A and two using Library B—per strategy.

All tracks used biotinylated recombinant human BCMA-rFc (cat. no. 10620-H03H-B, Sino Biological, Eschborn, Germany) as the target antigen. All incubation and wash steps were performed at RT with eoe rotation. SA-coated paramagnetic beads (Dynabeads M-280 Streptavidin, cat. no. 11205D, Invitrogen, Thermo Fisher Scientific, Waltham, MA, USA) were washed twice with PBS before use. To avoid unspecific binders, all tubes were pre-blocked with 1% (w/v) BSA in PBS-T. Phage solutions was pre-incubated for 30 min with 0.1% (w/v) BSA and beads, to remove phages carrying binders against SA.

Solid-phase selection strategy tracks (immobilisation of target antigen prior to incubation with phage): biotinylated human BCMA-rFc (140 nM (cycle 1), 40 nM (cycle 2) and 25 nM (cycle 3)) was immobilised on 0.5 mg SA-coated paramagnetic beads (Dynabeads M-280 Streptavidin, cat. no. 11205D, Invitrogen Thermo Fisher Scientific, Waltham, MA, USA) for 1 h, followed by blocking for 30 min with 1 % (w/v) BSA in PBS-T. Phage solutions were added to the immobilised and blocked biotinylated human BCMA-rFc and incubated for 2 h. SA-beads were washed with PBS-T for a total of 5 min (cycle 1), 10 min (cycle 2) or 20 min (cycle 3).

Liquid-phase selection strategy tracks (incubation of target antigen with phage in solution before immobilisation): phage solutions were incubated with biotinylated human BCMA-rFc (140 nM (cycle 1), 40 nM (cycle 2) and 25 nM (cycle 3)) for 2 h in cycles 1 and 3, or 1.5 h in cycle 2. Phage antigen complexes were captured by incubation with 0.5 mg SA-beads for 30 min. SA-beads were washed with PBS-T for a total of 5 min (cycle 1), 10 min (cycle 2) or 20 min (cycle 3).

Liquid-phase selection strategy with off-rate competition tracks: phage solutions were incubated with biotinylated human BCMA-rFc (30 nM (cycle 1) and 10 nM (cycles 2–3)) and incubated for 2 h in cycles 1 and 3, or 1.5 h in cycle 2. 10x molar excess of non-biotinylated human BCMA (BCMA-His,

10620-H08H, Sino Biological, Eschborn, Germany) was added and incubated for an additional 1 h. Phage antigen complexes were captured by incubation with 0.5 mg SA-beads for 30 min. SA-beads were washed with 100 nM non-biotinylated human BCMA in PBS-T for 4 min.

For all tracks, the final wash volumes were transferred to new tubes to remove sticky binders attached to the tube walls. Two tracks per selection strategy (one using Library A and one using Library B) used incubation with 0.5 M acetic acid, pH 2.8 for 15 min for elution, followed by neutralisation with an equal volume of 1 M Tris-HCl, pH 8. These eluted phages were later amplified using M13K07 helper phage (after cycle 1–2). For one track per selection strategy (using Library B), elution was conducted by incubating with 0.25 mg/ml trypsin (Gibco Life Technologies) in TBS-T (TRIS buffered saline, 0.1 % (v/v) Tween-20) supplemented with 1 mM $CaCl_2$ for 30 min. These eluted phages were later amplified using KM13 helper phage (after cycle 1–2).

Following selection cycles 1–2, helper phage M13K07 or KM13 were used to amplify new phage stocks in *E. coli* XL-1 Blue cells (Agilent, Santa Clara, CA, USA), for use as input phage in the subsequent selection cycle. Phage stock titres were measured by infecting *E. coli* XL-1 Blue and performing spot titration. Colony PCR was used to assess the percentage of infected colonies carrying phagemids with the correct affibody insert size.

1.5. Specificity analyses by SPR

Cross-reactivity studies were conducted using a Biacore T200 instrument (Cytiva, Uppsala, Sweden). The protein ligands were immobilised to a Series S CM5 sensor chip (Cytiva, Uppsala, Sweden) using amine coupling, with each chip containing one flow cell to be used as a reference cell (activated and deactivated). The ligands were: murine BCMA (Mouse BCMA / TNFRSF17 Protein, Fc Tag, cat. no. BCA-M5258, Acro Biosystems, Newark, DE, USA); rhesus macaque BCMA (Recombinant Rhesus BCMA Protein (hFc Tag), cat. no. 90103-C02H, Sino Biological, Eschborn, Germany); marmoset BCMA (HEK 293 expressed recombinant protein, corresponding to residues 1–52 of Uniprot entry F7IJE9, with a C-terminal human Fc tag, Mammalian transient expression service, Custom Recombinant Protein Expression Services, Sino biological, Eschborn, Germany), human BCMA-rFc (cat. no. 10620-H15H, Sino Biological, Eschborn, Germany); and human TACI (Human TACI /

TNFRSF13B Protein, His Tag, cat. no. TAI-H52H3, Acro Biosystems, Newark, DE, USA). 200 nM of 1-E6 (affibody-His_6 format) was injected over the flow cells. As a reference, 200 nM human APRIL (R&D systems, Minneapolis, MN, USA) was injected over the same flow cells. PBS-T was used as the running buffer and sample buffer, and the flow cells were regenerated with 10 mM HCl.

1.6. Fluorophore-labelling of 1-E6 homodimer

BCMA-binding clone 1-E6 was subcloned (In-Fusion HD Cloning Kit, TakaraBio, Gothenburg, Sweden) as a homodimer with a C-terminal His_6 tag (1-E6-1-E6-His_6) and produced in *E. coli* as described in the main text. Harvested cells were lysed by sonication and the resulting lysate supernatant was heat treated at 96 °C for 7 min. The extracted, heat-treated protein was purified under native conditions using HisPur Cobalt IMAC Resin (cat. no. 89966, Thermo Scientific, Waltham, MA, USA). Following purification, buffer exchange, absorbance measurement and SDS-PAGE analysis were performed as described in the main text. The purified protein was labelled overnight, eoe at RT, with AF647 fluorophore (Alexa Fluor 647 NHS Ester (Succinimidyl Ester), cat. no. A20006, Invitrogen, Thermo Fisher Scientific, Waltham, MA, USA) according to the manufacturer's instructions. Excess, unconjugated label was removed by desalting (NAP-5, cat. no. 17-0853-02, Cytiva, Uppsala, Sweden). Approximate sizes of non-labelled and AF647-labelled 1-E6 homodimer were compared by SDS-PAGE. MALDI mass spectrometry was used to determine the degree of labelling (DOL) of AF647-labelled 1-E6. Binding was compared using SPR (Biacore 8K, Cytiva, Uppsala, Sweden), where human BCMA-rFc was immobilised using amine coupling and 200 nM of non-labelled and AF647-labelled 1-E6 homodimer were injected. PBS-T was used as the running buffer and sample buffer, and the flow cells were regenerated with 10 mM HCl. Additional polishing was done by RP-HPLC, followed by analyses by mass spectrometry (Agilent 1290 UltraPerformance Liquid Chromatograph/Quadrople Time of Flight (Q-ToF) mass spectrometry detector, Agilent, Santa Clara CA, USA), SEC (Superdex 75 10/300GL column (Cytiva, Uppsala, Sweden) on an Agilent 1100 HPLC system (Agilent, Santa Clara, CA, USA)), and UV spectrophotometry at 280 and 650 nm (NanoDrop Spectrophotometer, Thermo Fisher Scientific, Waltham, MA, USA).

2. Supplementary figures

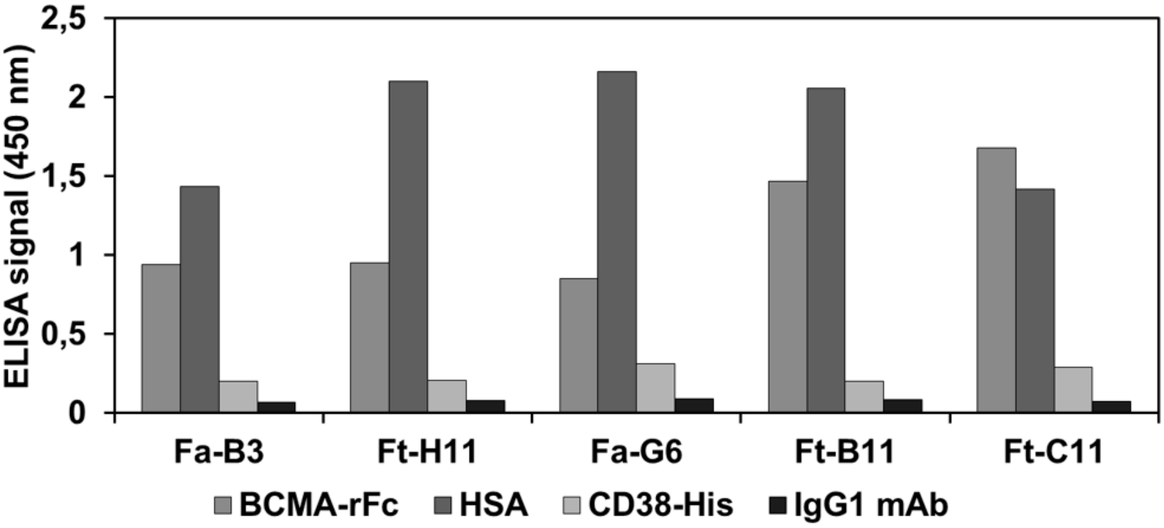

Figure S1. Monoclonal phage ELISA for five representative clones. ELISA plate wells were coated with either 5 µg/ml target antigen human BCMA Rabbit Fc fusion (BCMA-rFc) or control antigens: 15 µg/ml human serum albumin (HSA); 10 µg/ml IgG1 mAb; or 5 µg/ml unrelated control antigen (human CD38-His). Phage preparations from randomly picked clones obtained from the final selection cycle output were produced monoclonally and analysed for their binding to the target antigen and control antigens. HRP-conjugated anti-M13 phage antibody and TMB substrate were used to detect binding of phage to coated antigens (ELISA signal) at 450 nm.

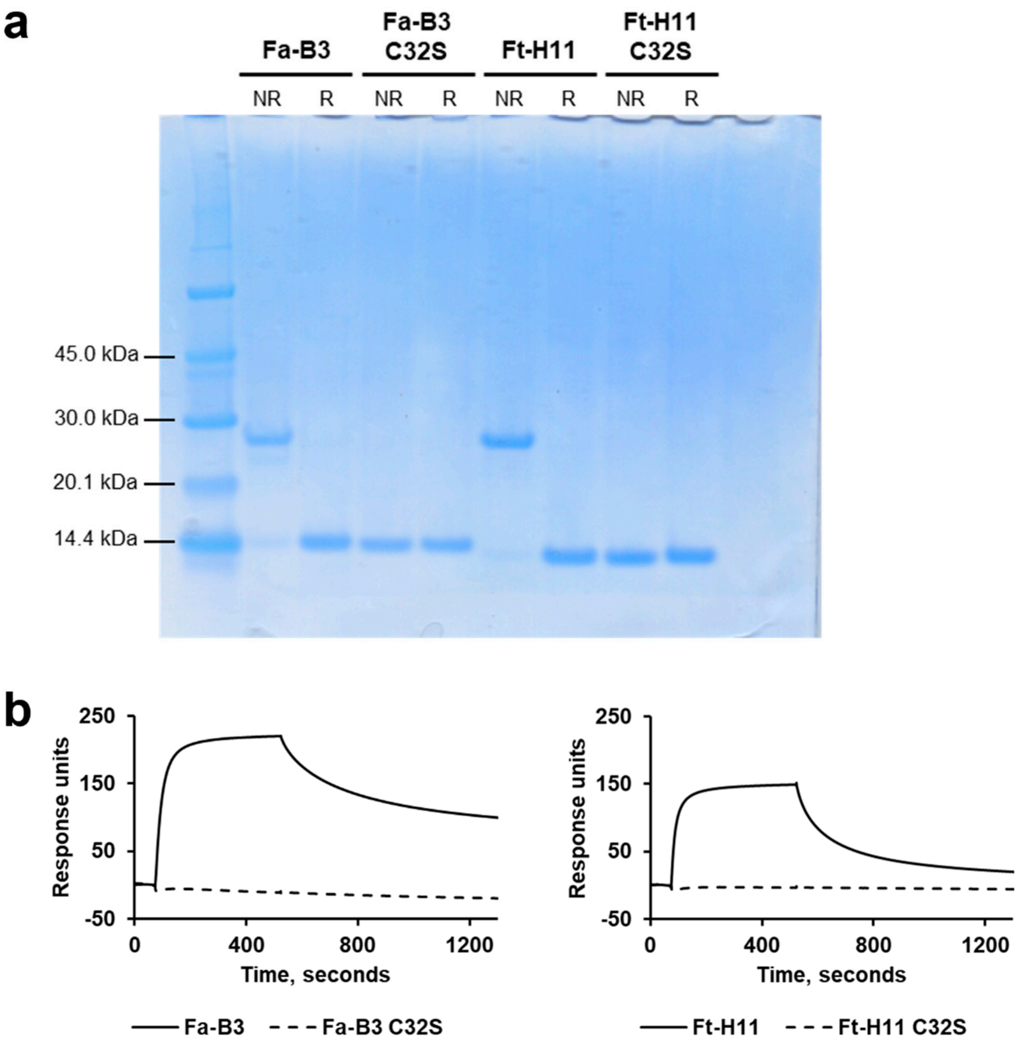

Figure S2. Analyses of cysteine-containing first-generation BCMA.binding clones Fa-B3 and Ft-B11 and corresponding cysteine-to-serine (C32S) mutation variants. (**a**) SDS-PAGE analysis of clones Fa-B3 and Ft-H11 under non-reducing and reducing conditions confirmed the presence of S-S-bridged homodimers. C32S mutation variants did not demonstrate any formation of dimers under non-reducing conditions. (**b**) SPR analysis showed loss of binding to immobilised human BCMA Rabbit Fc fusion (BCMA-rFc) for C32S mutation variants of Fa-B3 and Ft-H11.

a

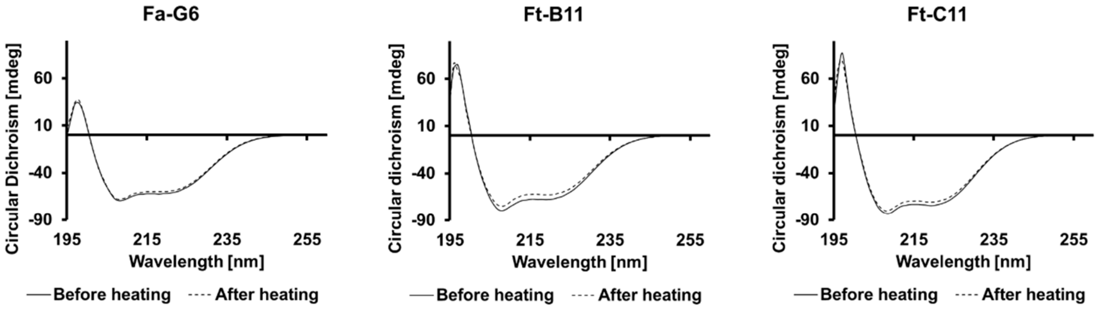

b

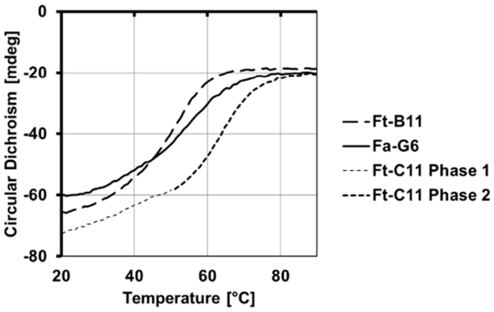

c

Clone	Approximate T_m (°C)
Ft-B11	49
Fa-G6	52
Ft-C11 (Phase 1)	36
Ft-C11 (Phase 2)	64

Figure S3. Circular dichroism spectroscopy analysis of first-generation BCMA-binding affibodies. (**a**) Secondary structure content of first-generation BCMA-binding affibodies Ft-B11, Fa-G6 and Ft-C11. Ellipticity in millidegrees (mdeg) of affibodies (0.5 mg/ml) was measured at wavelengths 195–260 nm at 20 °C (before heating), followed by a second measurement after heating the samples to 90 °C and then cooling them to 20 °C (after heating). (**b**) Thermal denaturing profiles (heating from 20 °C to 90 °C, at a rate of 5 °C/min). Clone Ft-C11 demonstrated biphasic melting behaviour, marked as Phase 1 and Phase 2 in the figure. (**c**) Estimated melting temperatures (Tm) based on the obtained thermal denaturing profiles. Two Tm values are estimated for clone Ft-C11, one for each of Phase 1 and Phase 2.

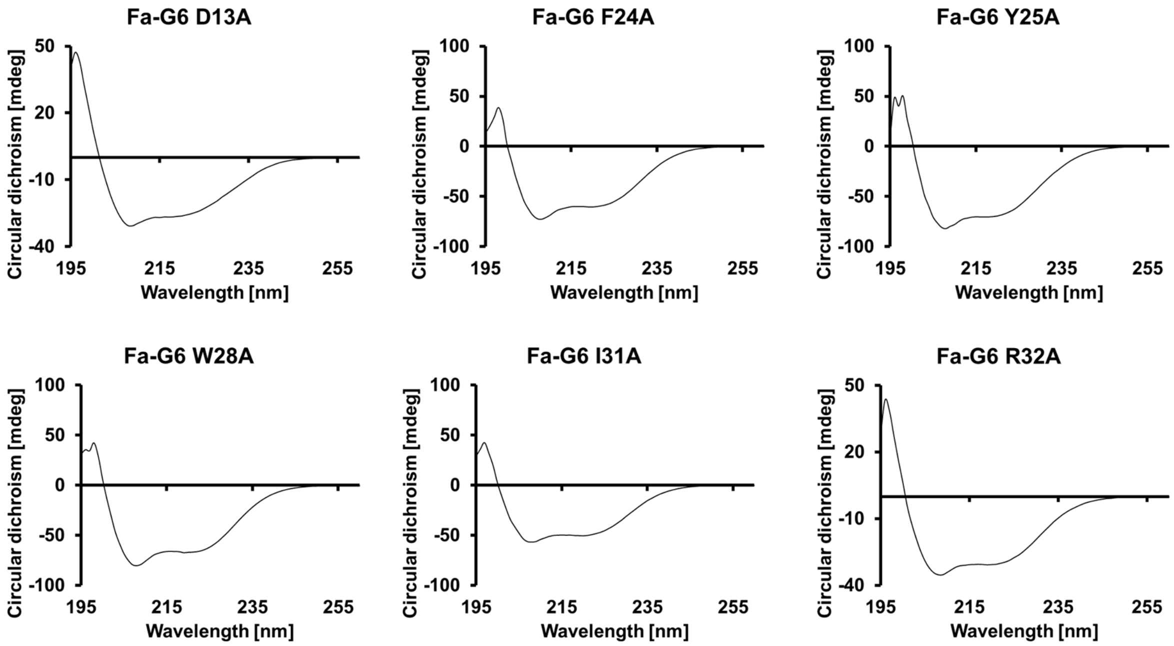

Figure S4. Circular dichroism spectroscopy analysis of alanine variants of Fa-G6. Secondary structure content of Fa-G6 alanine variants D13A, F24A, Y25A, W28A, I31A and R32A (0.5 mg/ml) was measured by recording the ellipticity in millidegrees (mdeg) at wavelengths 195–260 nm at 20 °C.

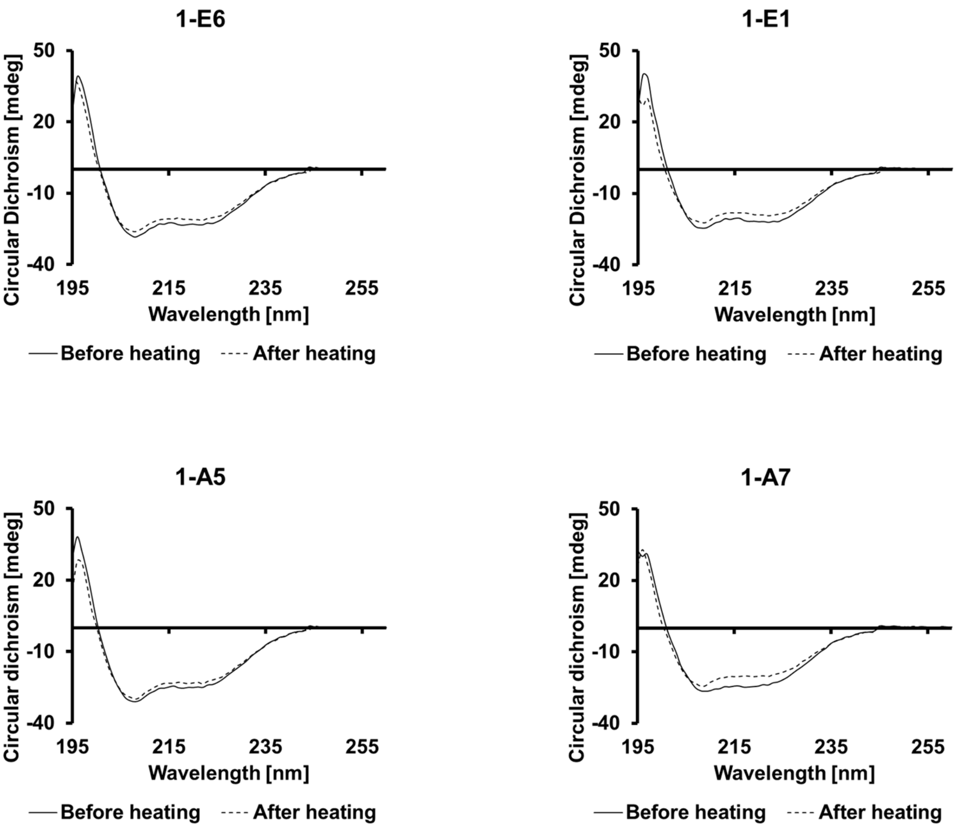

Figure S5. Secondary structure content of second-generation BCMA-binding affibodies 1-E6, 1-E1, 1-A5, and 1-A7. Ellipticity in millidegrees (mdeg) of affibodies (0.2 mg/ml) was measured at wavelengths 195–260 nm at 20 °C (before heating), followed by a second measurement after heating the samples to 90 °C and then cooling them to 20 °C (after heating).

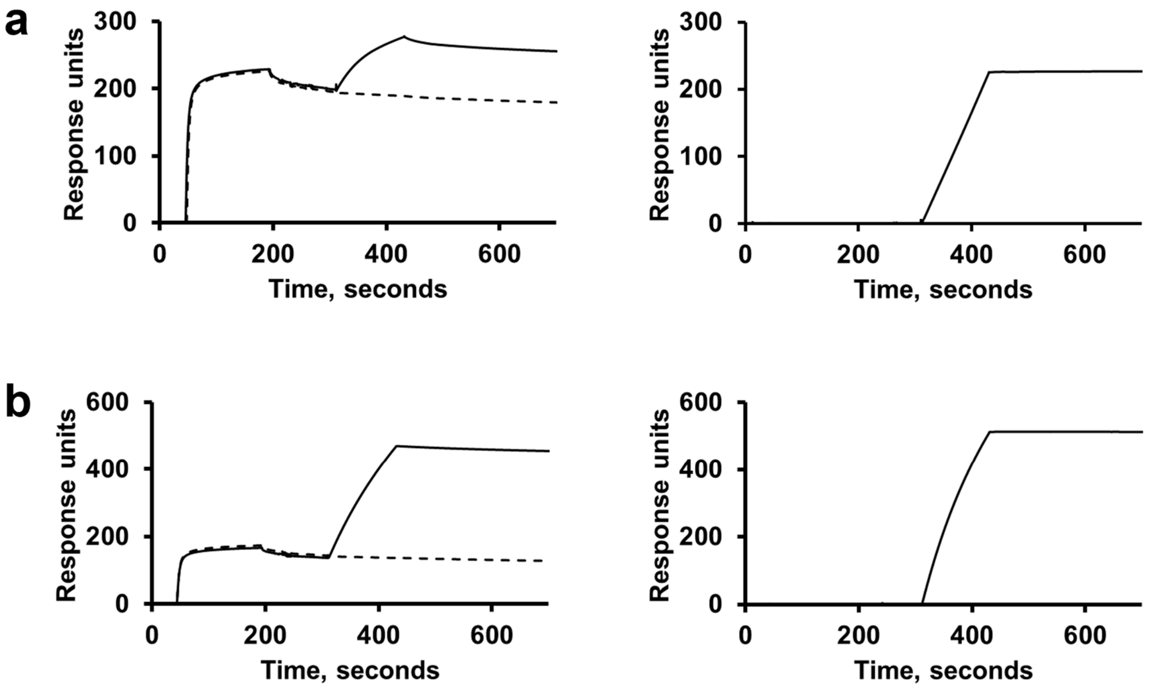

Figure S6. SPR response signals from epitope mapping analyses. The left panel shows 1 µM 1-E6 affibody injected over immobilised human BCMA-rFc in a first injection, followed by either running buffer (dashed line) or BCMA-binding analyte (solid line). The right panel shows a first injection of running buffer followed by an injection of BCMA-binding analyte. The BCMA-binding analytes were (**a**) 100 nM human APRIL and (**b**) 25 nM belantamab.

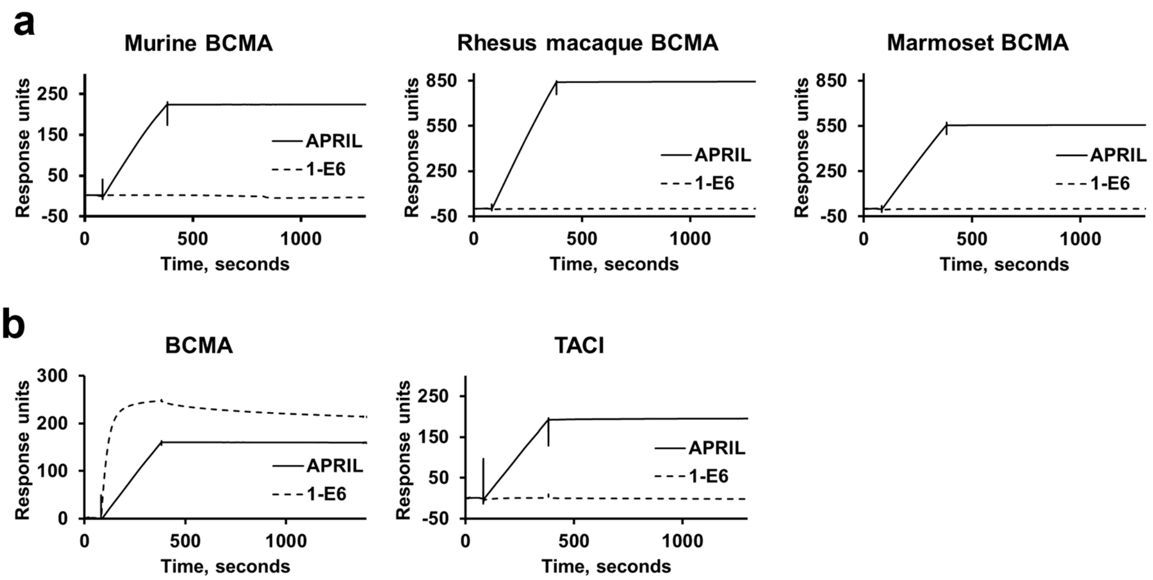

Figure S7. Cross-reactivity studies of the BCMA-binding 1-E6 affibody. (**a**) Analysis of 1-E6 binding to BCMA from murine, rhesus macaque and marmoset species. No binding could be detected to any of the tested BCMA homologues. Human APRIL showed binding to all three species homologues. (**b**) Analysis of 1-E6 binding to human BCMA and TACI. No binding to TACI could be detected for 1-E6, whereas human APRIL bound to both immobilised ligands.

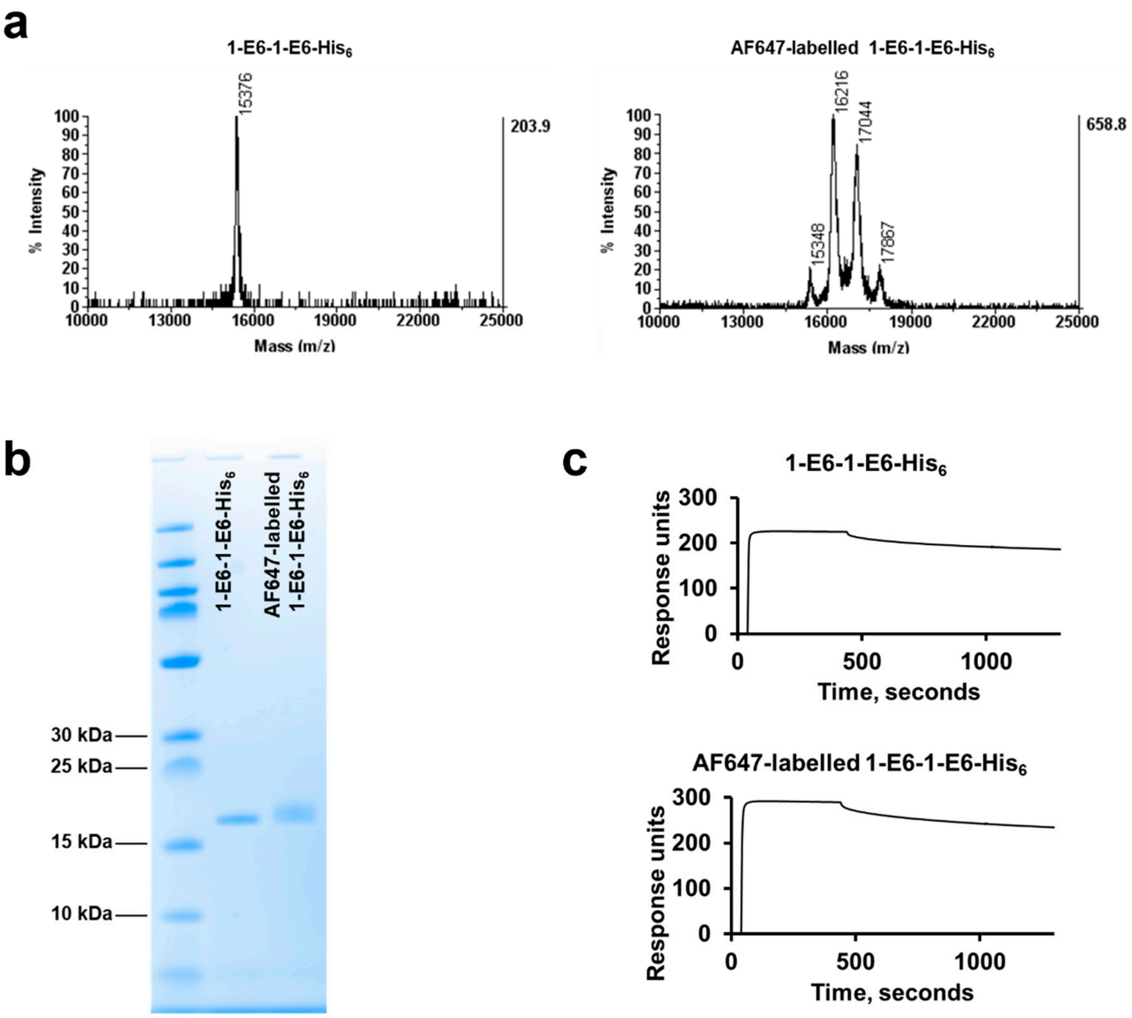

Figure S8. Fluorophore-labelling of 1-E6 affibody. (**a**) The recombinantly produced and purified 1-E6 homodimer (1-E6-1-E6-His$_6$, theoretical molecular weight (MW) 15384 Da) was labelled with AF647 fluorophore (Alexa Fluor 647, NHS Ester bioconjugation labelling) overnight. Following removal of excess unconjugated fluorophore dye, the resulting conjugated preparation was analysed by MALDI-MS, which showed the number of labels to range between 0–3 fluorophore labels per affibody molecule (average mass increase 814 m/z for each additional label). (**b**) SDS-PAGE gel of unlabelled and AF647-labelled 1-E6 affibody homodimer. Labelled product migrated larger and with a smeared band, due to the heterogeneity in the labelled preparation. (**c**) Unlabelled and AF647-labelled 1-E6 affibody homodimer were injected over immobilised human BCMA-rFc, which showed that the labelled preparation overall was not affected in its binding to BCMA following fluorophore conjugation.

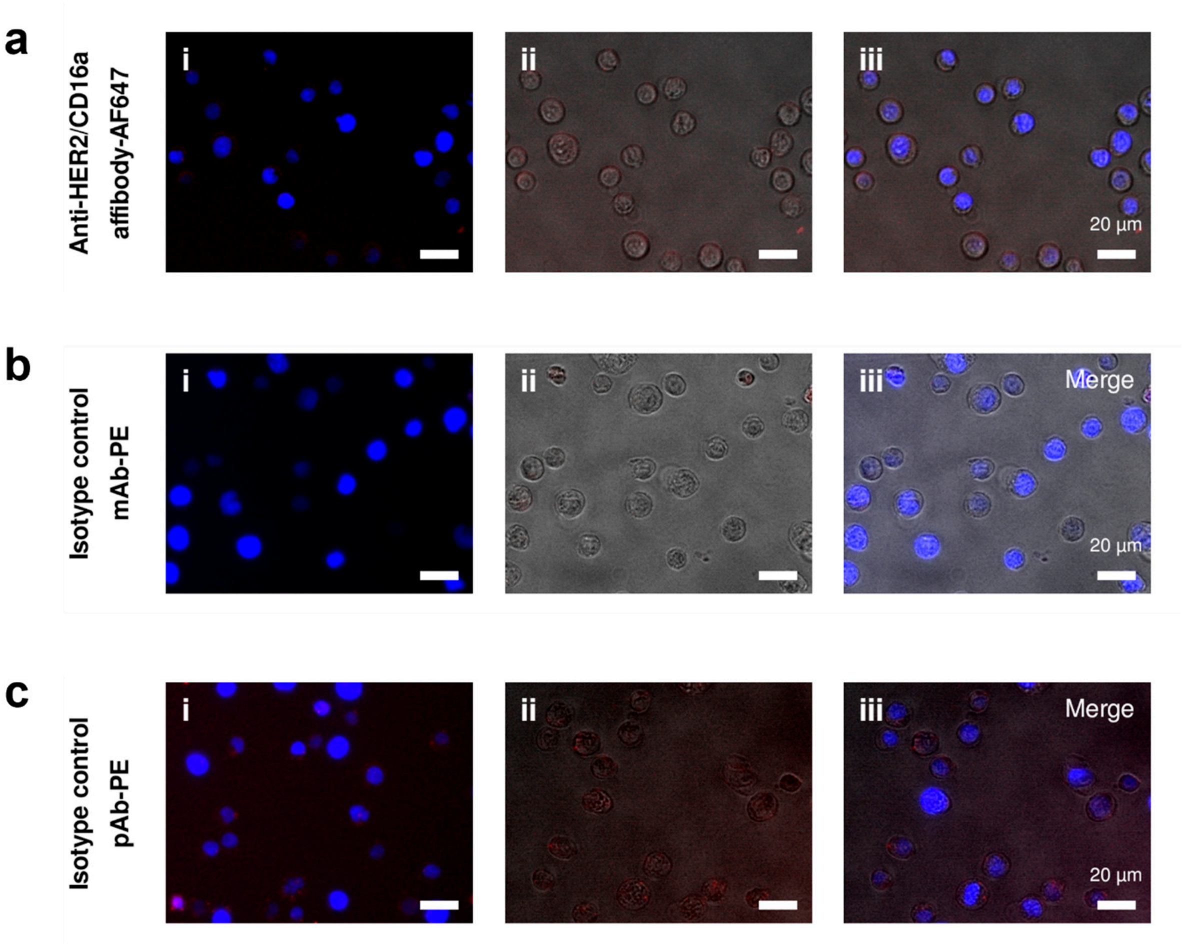

Figure S9. Representative fluorescence and brightfield microscopy images of MM.1s cells stained with control reagents. AF647-labelled anti-HER2/CD16a affibody control (red) (**a**) and PE-labelled isotype controls antibodies, monoclonal mouse IgG2a (red) (**b**) and polyclonal goat IgG (red) (**c**), show little to negligible binding of MM.1s cells. **a.i-c.i** show the fluorescence staining. In **a.ii-c.ii,** the fluorescence signal of each reagent is overlapped with an image acquired with brightfield microscopy, to visualise the shape of the cells. Lastly, a merge of **i** and **ii** is shown in **a.iii-c.iii**. Nuclei are stained with DAPI (blue). Scale bars: 20 µm.

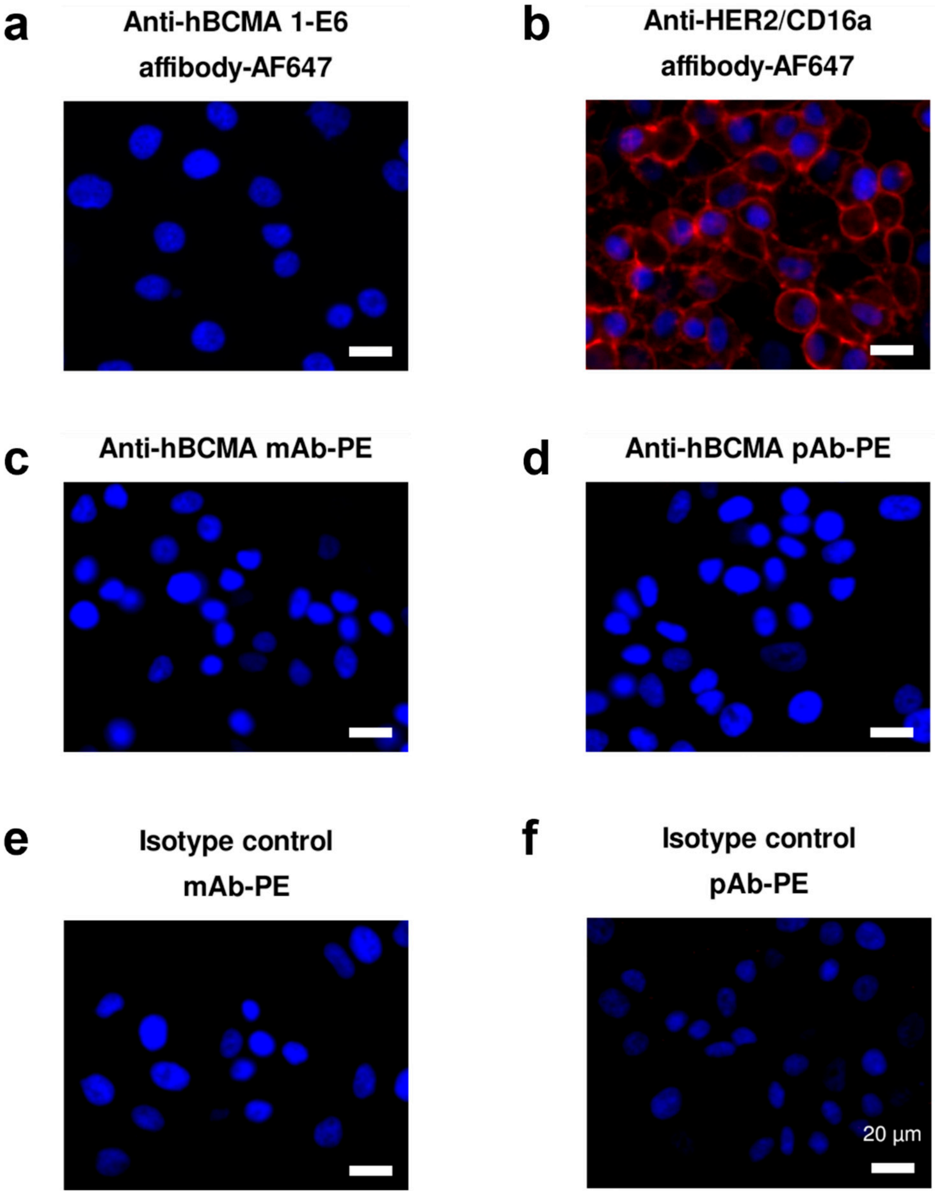

Figure S10. Representative fluorescence microscopy images of SKBR3 cells stained with affibody and antibody reagents. No binding could be observed for AF647-labelled 1-E6 affibody (1-E6-1-E6-His_6) (red) (**a**), BCMA-binding antibodies (red) (**c-d**), or corresponding isotype control antibodies (red) (**e-f**). (**b**) Anti-HER2/CD16a control affibody (red) demonstrated binding to the SKBR3 cells. Nuclei are stained with DAPI (blue). Scale bars: 20 µm.
