# Supplementary figures and images for: An Anti-BCMA Affibody Affinity Protein for Therapeutic and Diagnostic Use in Multiple Myeloma
Source: Int J Mol Sci. 2025 May 28;26(11):5186. doi: 10.3390/ijms26115186 (PMC12154184; doi:10.3390/ijms26115186)

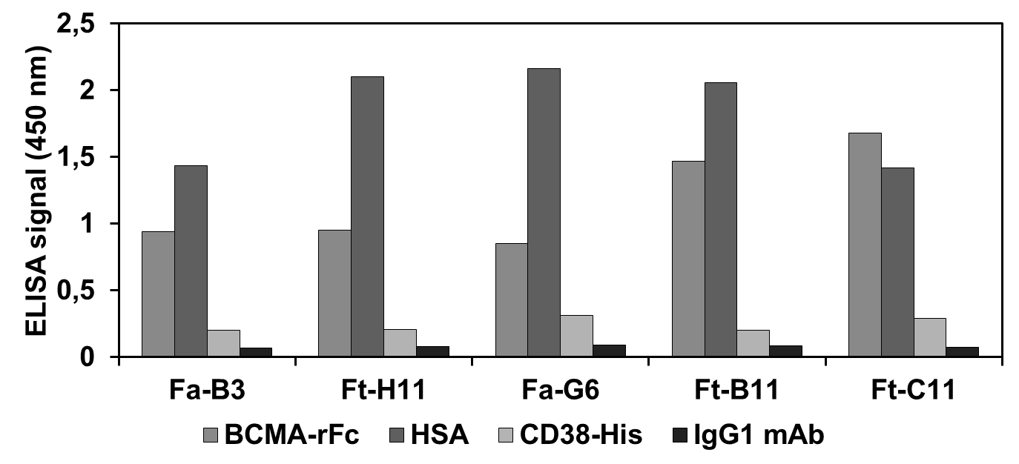

Supplement: Supplementary file 1 [file ijms-26-05186-s001.zip › S1.png]

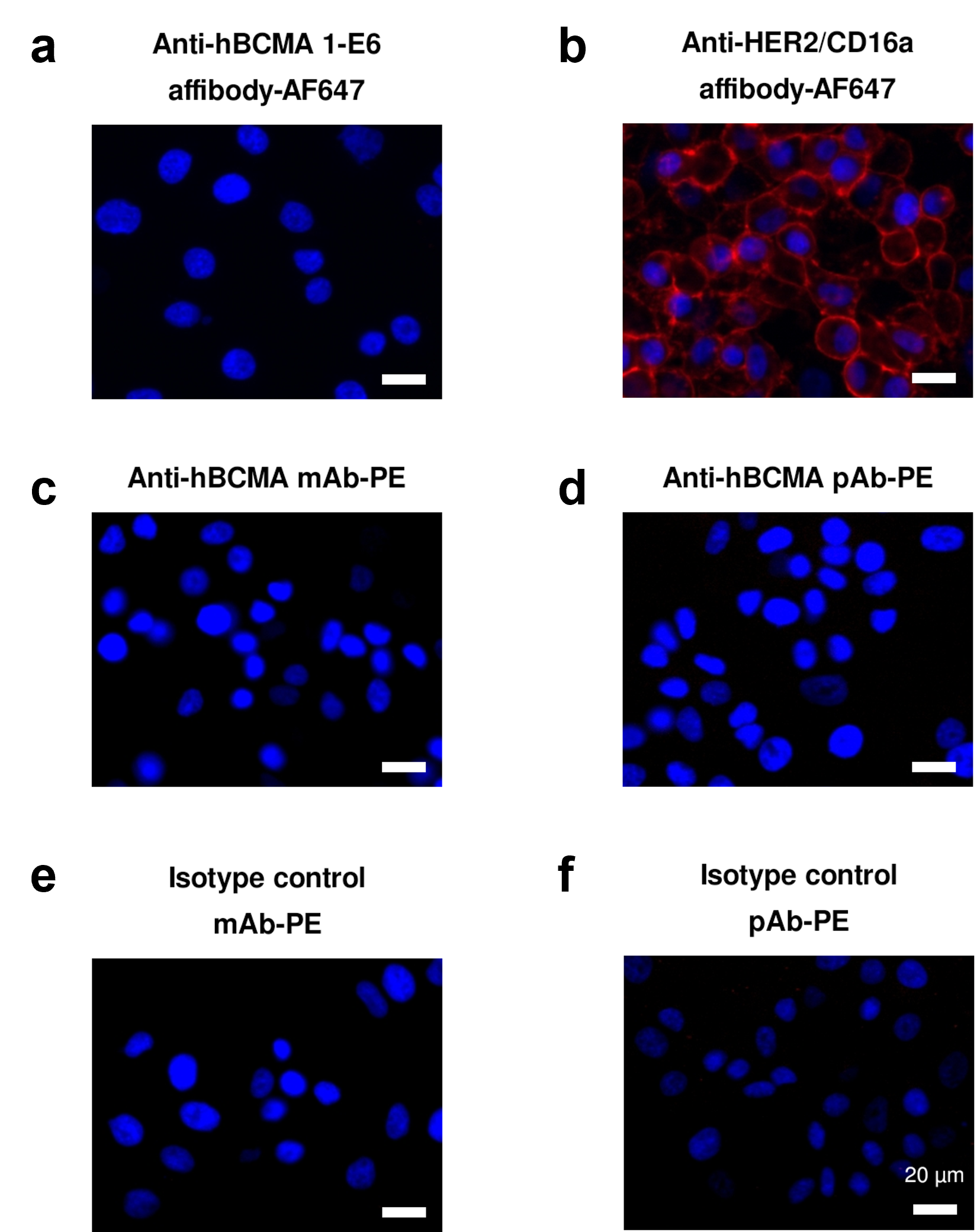

Supplement: Supplementary file 1 [file ijms-26-05186-s001.zip › S10.png]

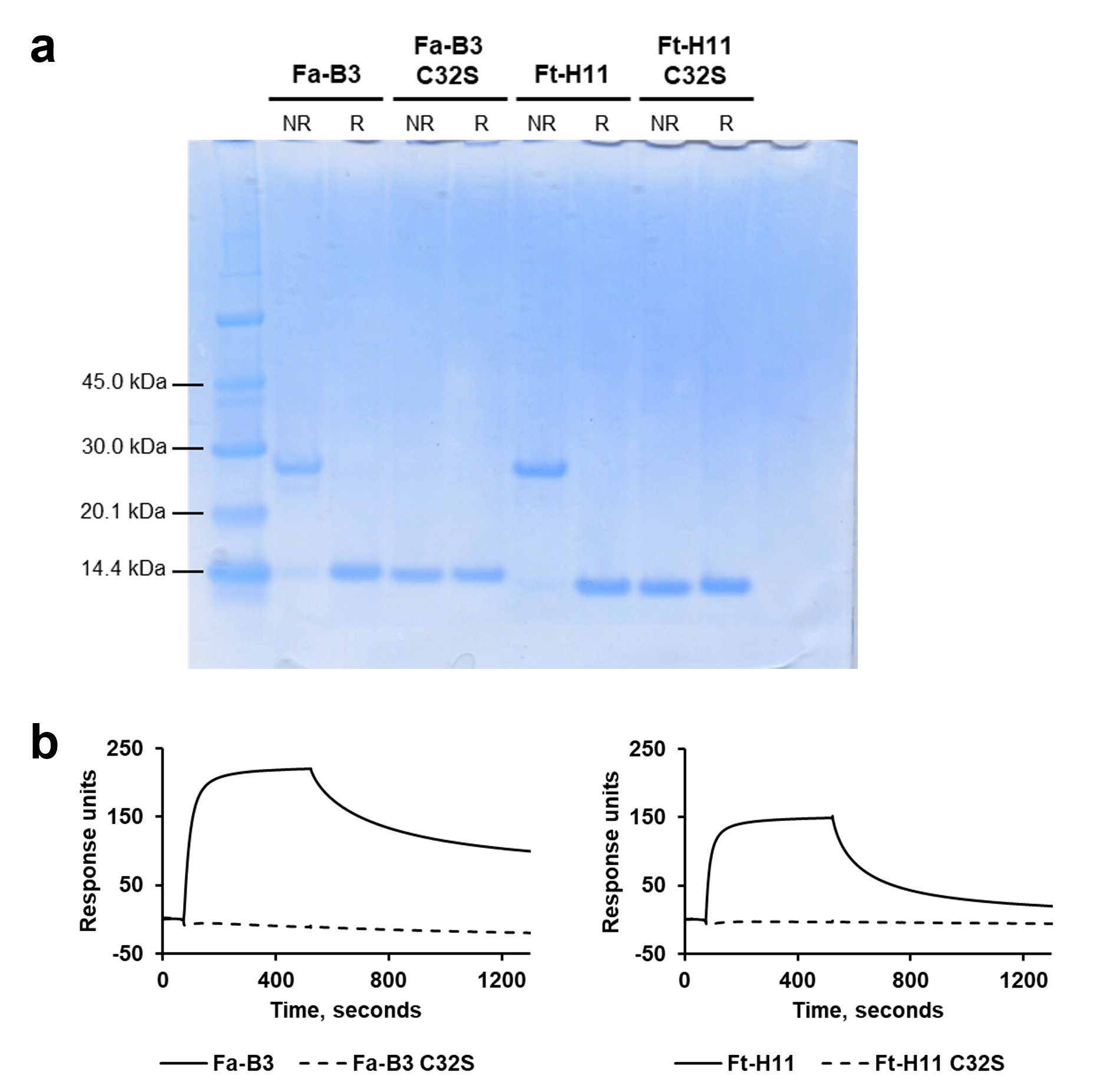

Supplement: Supplementary file 1 [file ijms-26-05186-s001.zip › S2.png]

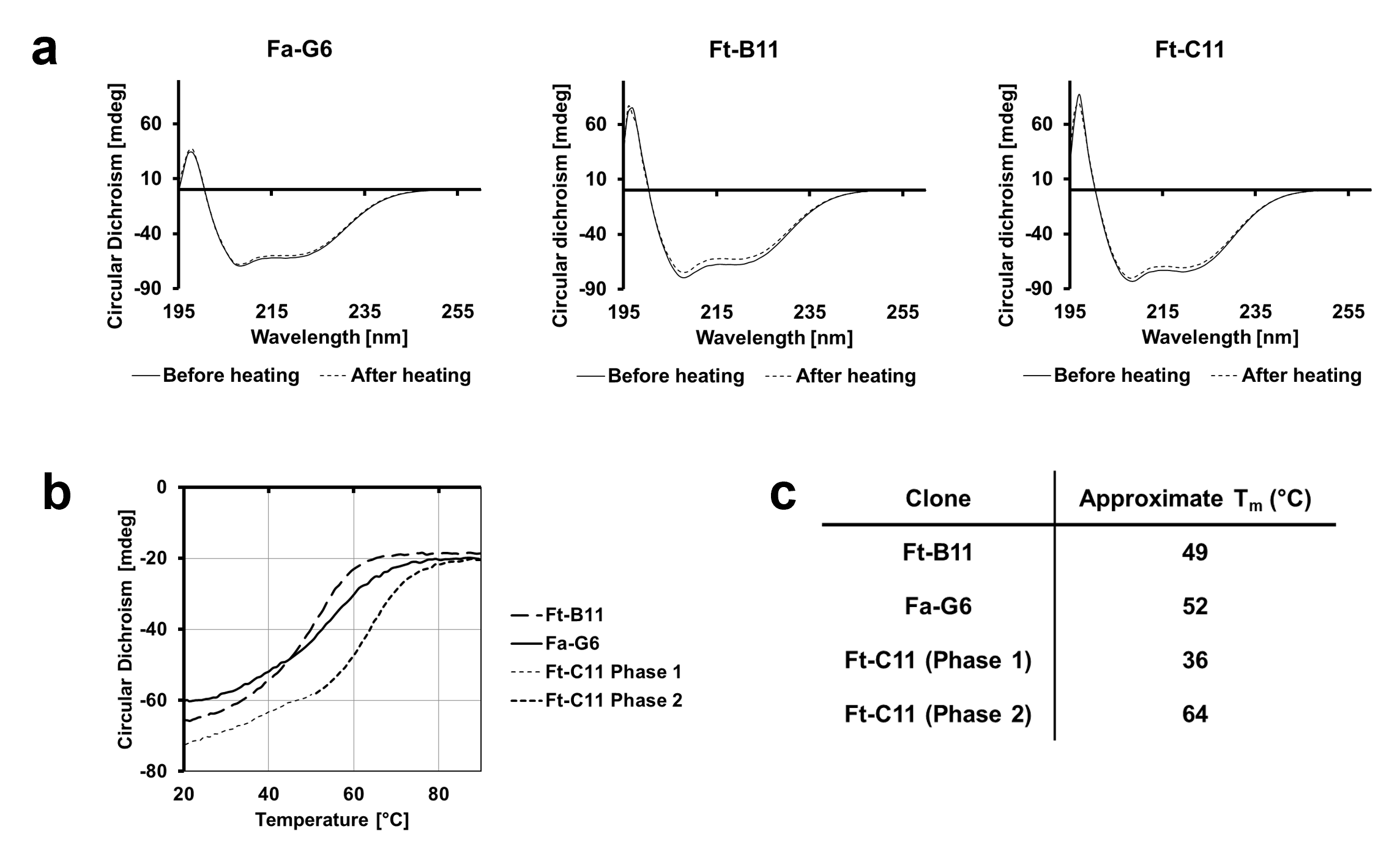

Supplement: Supplementary file 1 [file ijms-26-05186-s001.zip › S3.png]

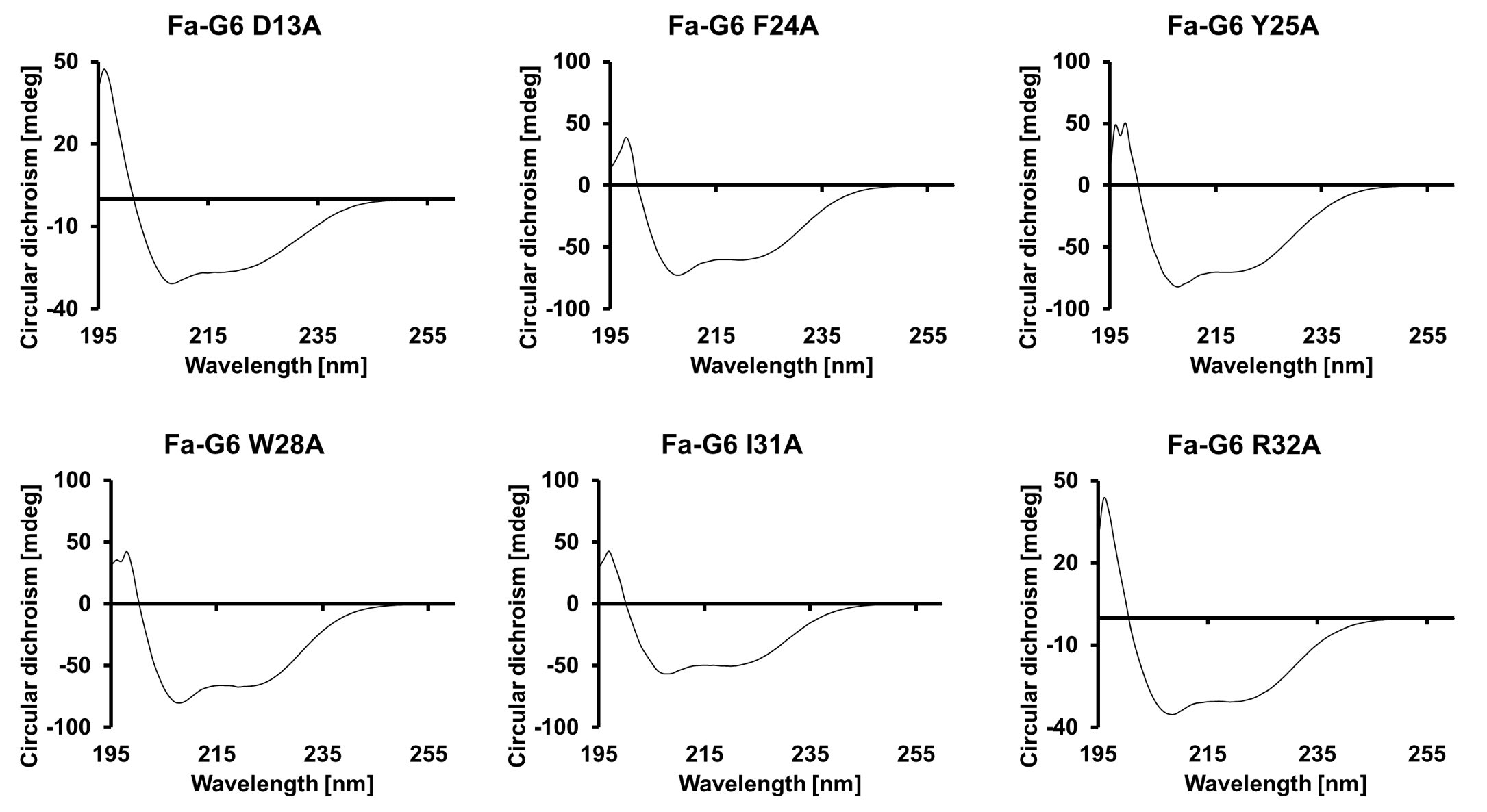

Supplement: Supplementary file 1 [file ijms-26-05186-s001.zip › S4.png]

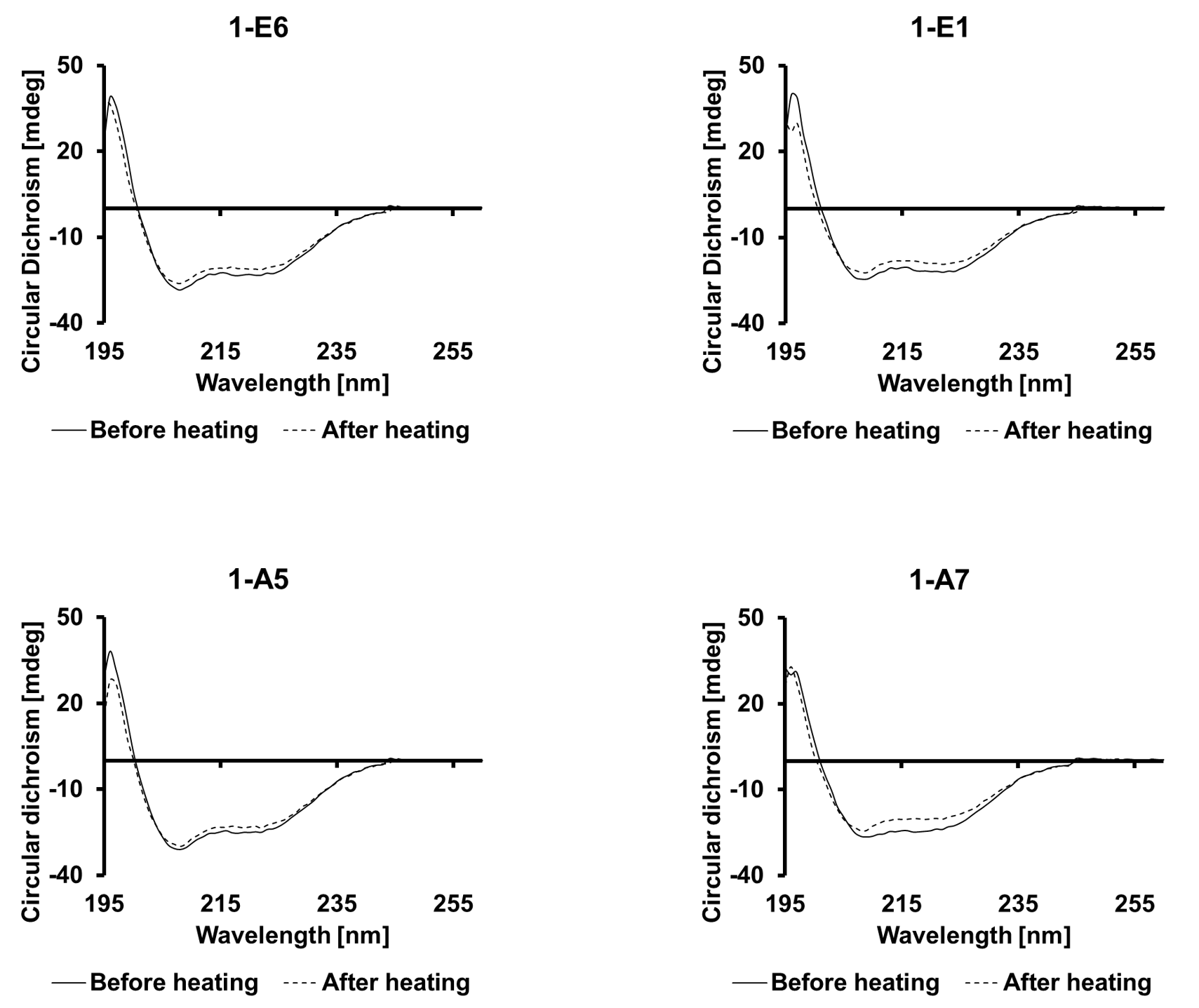

Supplement: Supplementary file 1 [file ijms-26-05186-s001.zip › S5.png]

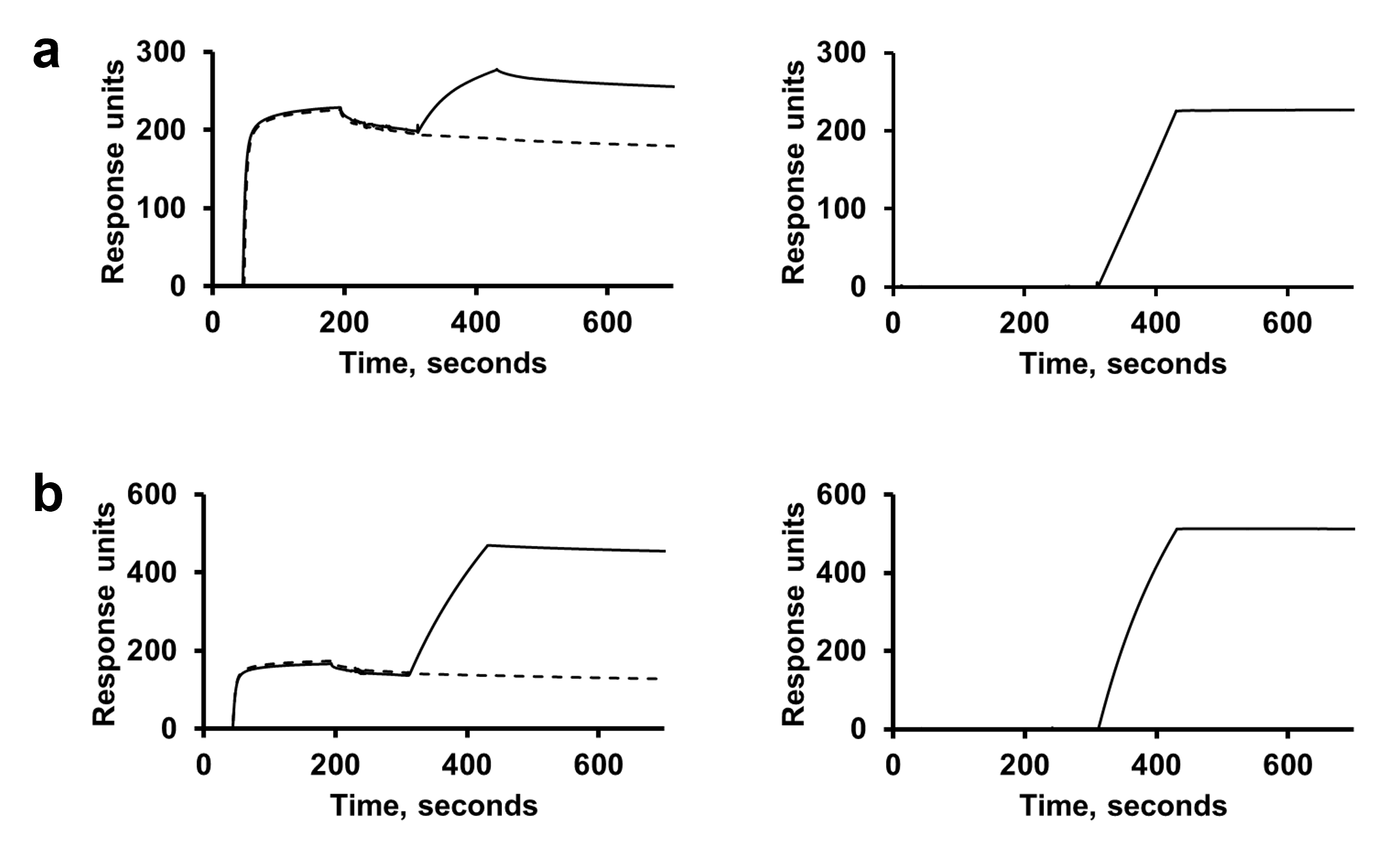

Supplement: Supplementary file 1 [file ijms-26-05186-s001.zip › S6.png]

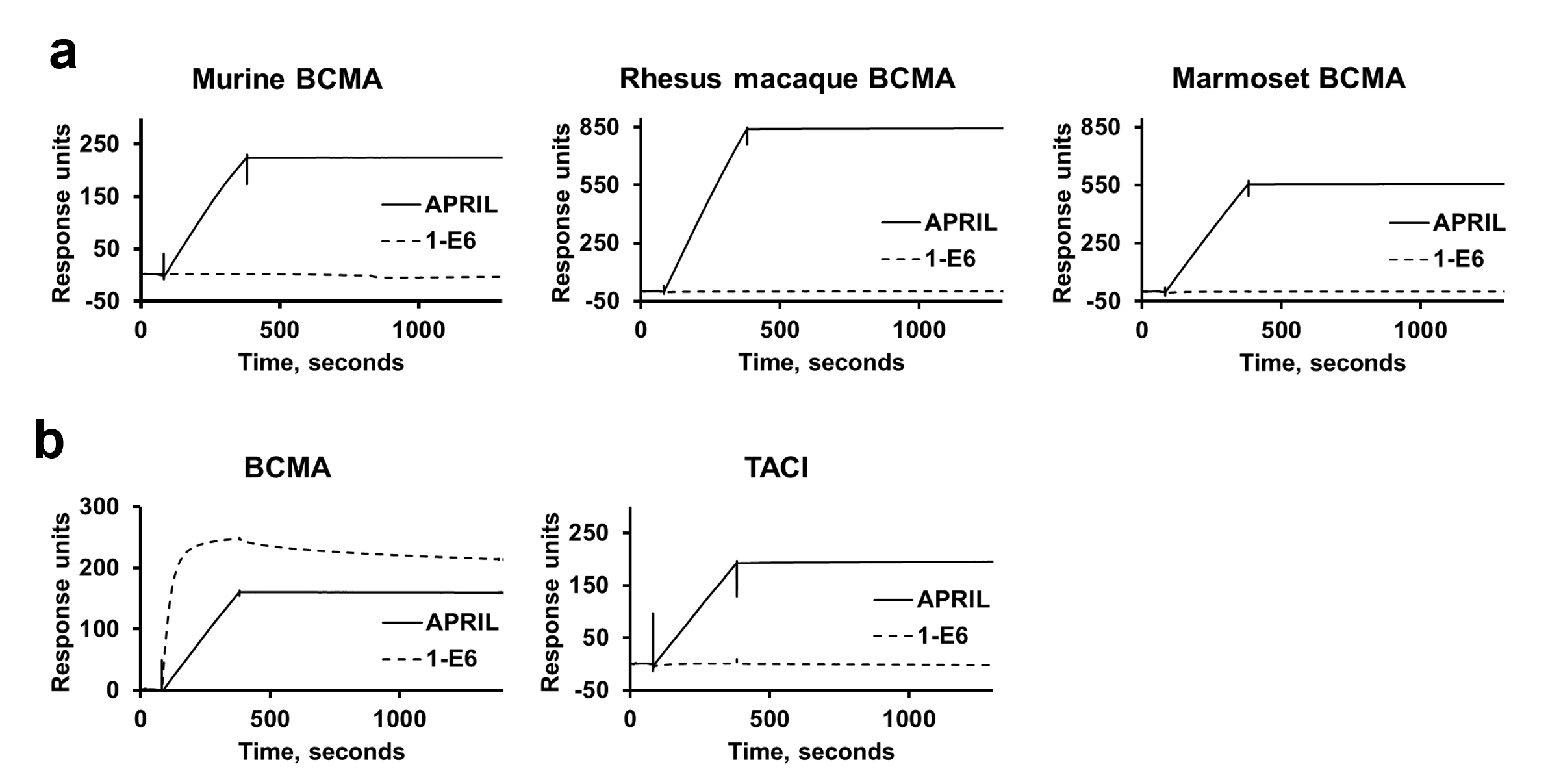

Supplement: Supplementary file 1 [file ijms-26-05186-s001.zip › S7.png]

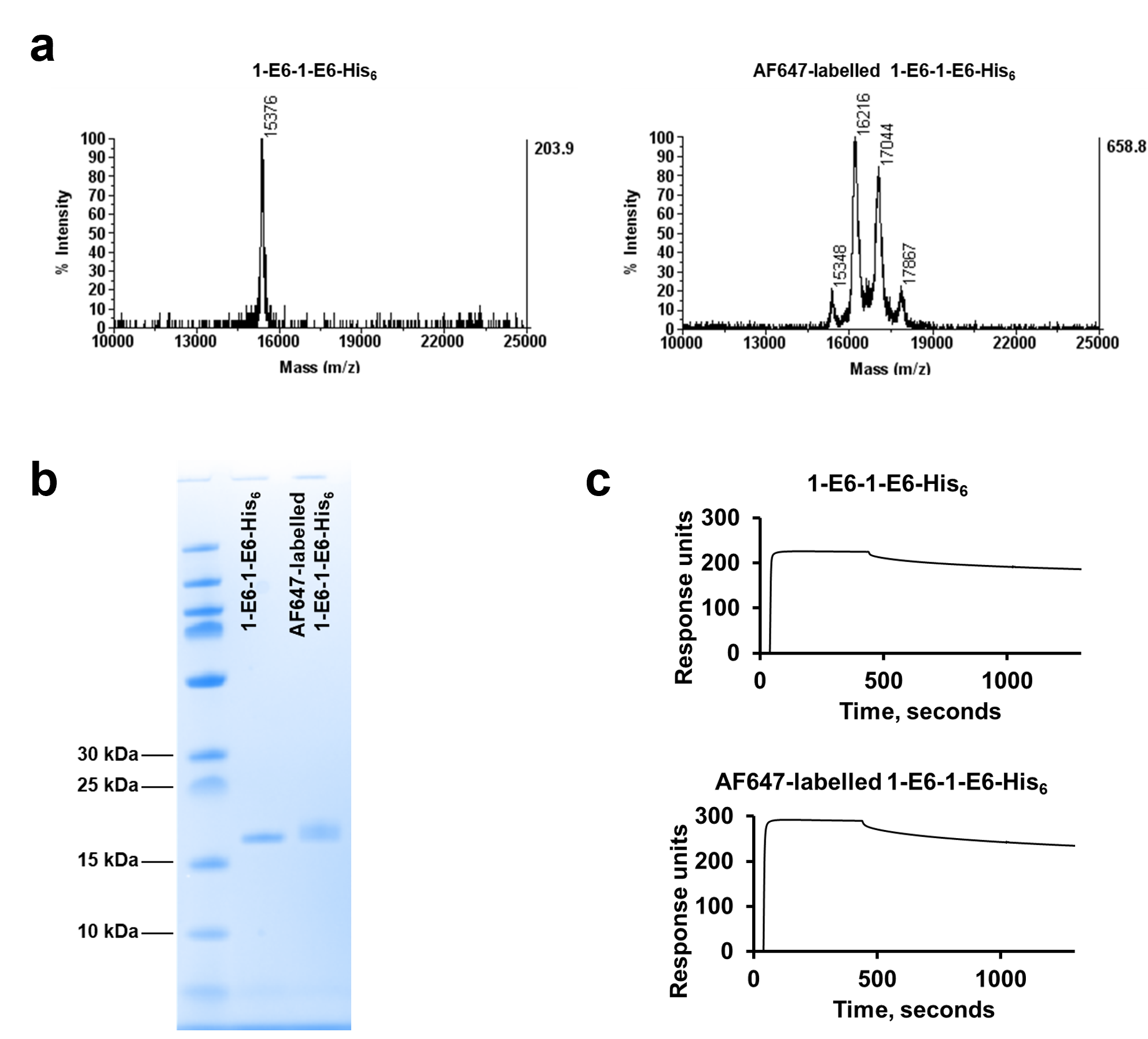

Supplement: Supplementary file 1 [file ijms-26-05186-s001.zip › S8.png]

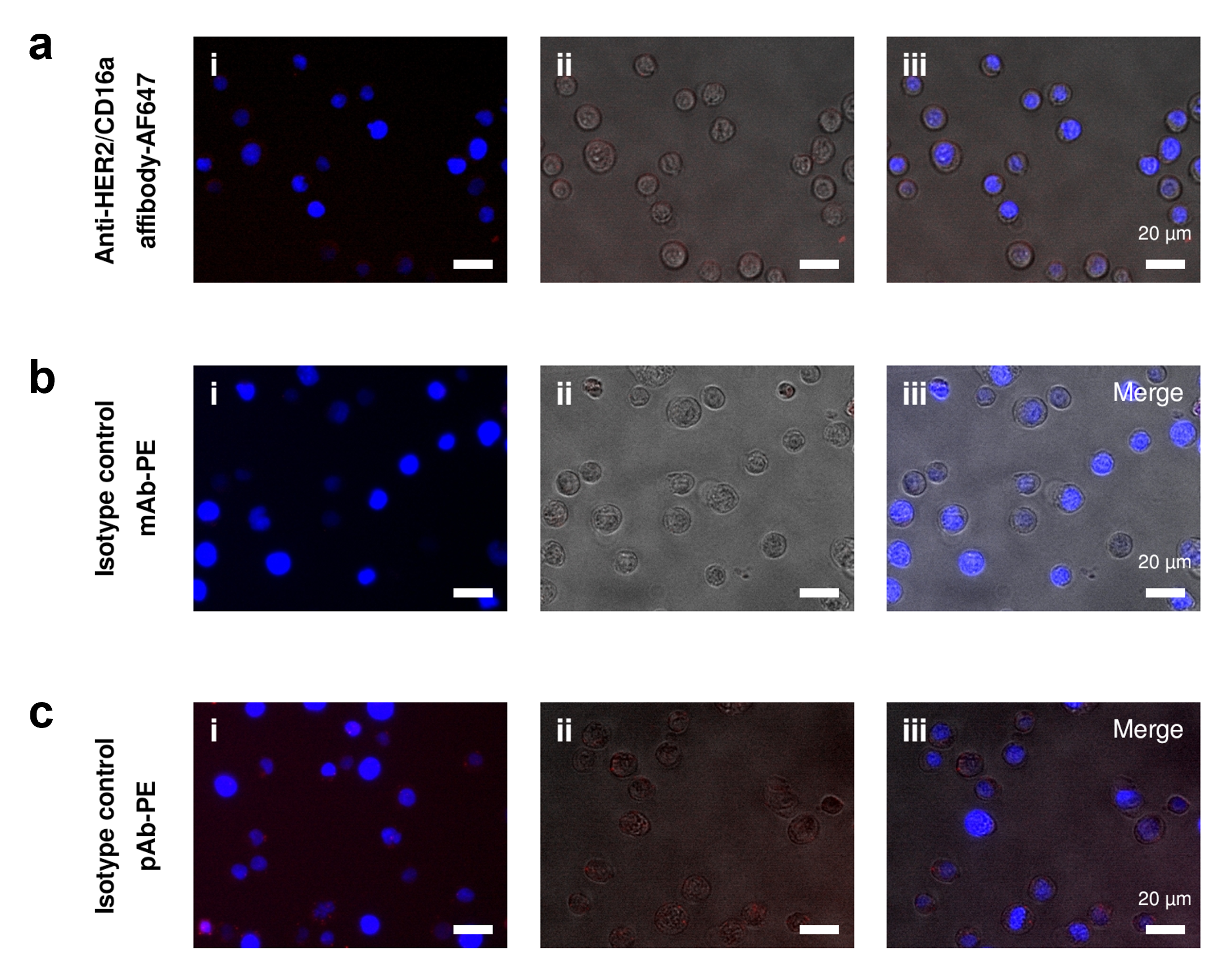

Supplement: Supplementary file 1 [file ijms-26-05186-s001.zip › S9.png]
